# Three‐State Thermochromic Smart Window for Building Energy‐Saving

**DOI:** 10.1002/advs.202416688

**Published:** 2025-03-20

**Authors:** Meiling Liu, Xiansheng Li, Wenshuo Zhang, Lanxin Li, Liang Li, Chengming Wang, Gang Pei, Bin Zhao, Chongwen Zou

**Affiliations:** ^1^ National Synchrotron Radiation Laboratory, School of Nuclear Science and Technology University of Science and Technology of China Hefei Anhui 230029 P. R. China; ^2^ Department of Thermal Science and Energy Engineering University of Science and Technology of China Hefei Anhui 230027 P. R. China; ^3^ Instruments' Center for Physical Science, Hefei National Research Center for Physical Sciences at the Microscale University of Science and Technology of China Hefei Anhui 230027 P. R. China

**Keywords:** energy‐saving, smart window, thermochromic, three‐state

## Abstract

Smart windows that dynamically regulate solar spectrum transmission to reduce energy consumption in heating, ventilation, and air conditioning systems are highly desirable. However, the limited amplitude of the regulation and narrow wavelength control often degrade the modulation performance of existing smart windows. To improve solar modulation and thermal management, here we propose a three‐state thermochromic smart window (TSSW) capable of modulation across the entire solar spectrum. The TSSW is mainly based on the unique phase transition properties of tungsten‐doped vanadium dioxide (W‐VO₂) and perovskite films, which can stepwise control the visible light and near‐infrared (NIR) transmittance separately, leading to the adaptive transitions between cold, warm, and hot states. Results indicate that the TSSW achieves a solar modulation rate of 23.5%, with indoor solar irradiance decreasing from 413.6 W/m^2^ in the cold state to 374.5 W/m^2^ in the warm state, and down to 189.1 W/m^2^ in the hot state. The simulation results show that the annual total energy demand can be reduced by up to 102.09 WJ/m^2^ in some typical regions. Compared to Low‐E glass and ordinary glass, this TSSW offers superior energy‐saving potential, making it an ideal solution for reducing building energy consumption.

## Graphical abstract

A unique three‐state thermochromic smart window (TSSW) featuring W‐VO₂ and perovskite adapts to cold, warm, and hot states, dynamically modulating visible and NIR transmittance across the solar spectrum for superior energy efficiency.

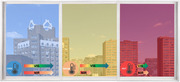

## 1 Introduction

Energy remains a critical global issue and a key focus in modern science and technology.^[^ ^1^, ^2^, ^3^ ^]^ In developed countries, buildings account for ≈40% of total energy consumption. For buildings, the attached windows always serve as the major conduits for energy exchange between indoor and outdoor environments, which makes them the least energy‐efficient building components.^[^ ^4^, ^5^ ^]^ Though ordinary glass windows provide sufficient daylighting, they also allow excessive solar radiation, causing significant indoor temperature fluctuations. Thus, it is necessary to use air conditioning facilities to keep the comfortable indoor temperature, increasing energy consumption.^[^ ^6^ ^]^ Accordingly, optimizing the building's energy efficiency and daylighting performance by regulating solar radiation transmission is essential.

In recent years, smart windows, which dynamically adjust spectral transmittance in response to environmental stimuli, have garnered significant attention and rapid development. Among these, thermochromic smart windows are particularly promising due to their passive response to temperature changes, requiring no external energy input. Various thermo‐responsive materials, including vanadium dioxide (VO₂),^[^ ^7^, ^8^, ^9^, ^10^ ^]^ hydrogels,^[^ ^11^, ^12^, ^13^, ^14^, ^15^ ^]^ ionic liquids,^[^ ^16^ ^]^ liquid crystals,^[^ ^17^, ^18^, ^19^ ^]^ and perovskites,^[^ ^6^, ^20^, ^21^ ^]^ have been successfully employed to regulate transmittance via different mechanisms. For example, VO₂ nanocrystals, ionic liquids, and perovskites regulate absorption via phase transitions, while hydrogels and liquid crystals modulate transmittance through phase separation and crystal orientation changes, respectively. Despite advances, conventional thermochromic smart windows have primarily focused on improving radiative transfer in a specific wavelength range, such as single‐band regulation for visible light or dual‐band regulation for the solar spectrum.^[^ ^12^, ^20^, ^22^ ^]^ These designs are often limited to a critical temperature and can only switch between two states (hot and cold). In the hot state, they minimize the transmittance of solar radiation to reduce heat gain, while in the cold state, they maximize the transmittance to increase solar heat. Although they can effectively reduce unwanted solar heat, they cannot dynamically and stepwise modulate visible and near‐infrared transmittance, nor can they control the heat entering the room according to different ambient temperatures, limiting their modulation capability. In fact, for the practical application of smart windows, visible light lighting and near‐infrared blocking/transmittance are both the required functions. Thus, achieving the separate and stepwise control of the near‐infrared and visible transmittance in the solar spectral range is highly desirable. However, there are few reports on the three‐state thermochromic smart windows for the stepwise modulation of visible and near‐infrared transmittance separately.

To address this limitation, we have developed a three‐state thermochromic smart window, which utilizes the different critical temperatures of W‐VO₂ and perovskite and can adaptively and gradually regulate visible and near‐infrared radiation. Experimental results demonstrate that the smart window can reversibly transition between three distinct states (cold, warm, and hot) in response to temperature changes, thereby achieving adaptive control over visible and near‐infrared light transmission. After structural optimization, the fabricated smart window achieved a visible light transmittance greater than 43.5% and a solar modulation rate of 23.5%, indicating promising potential for future energy‐saving applications.

## 2 Results and Discussion

### 2.1 Ideal Schematic of Three‐State Thermochromic Smart Window

**Figure 1:**
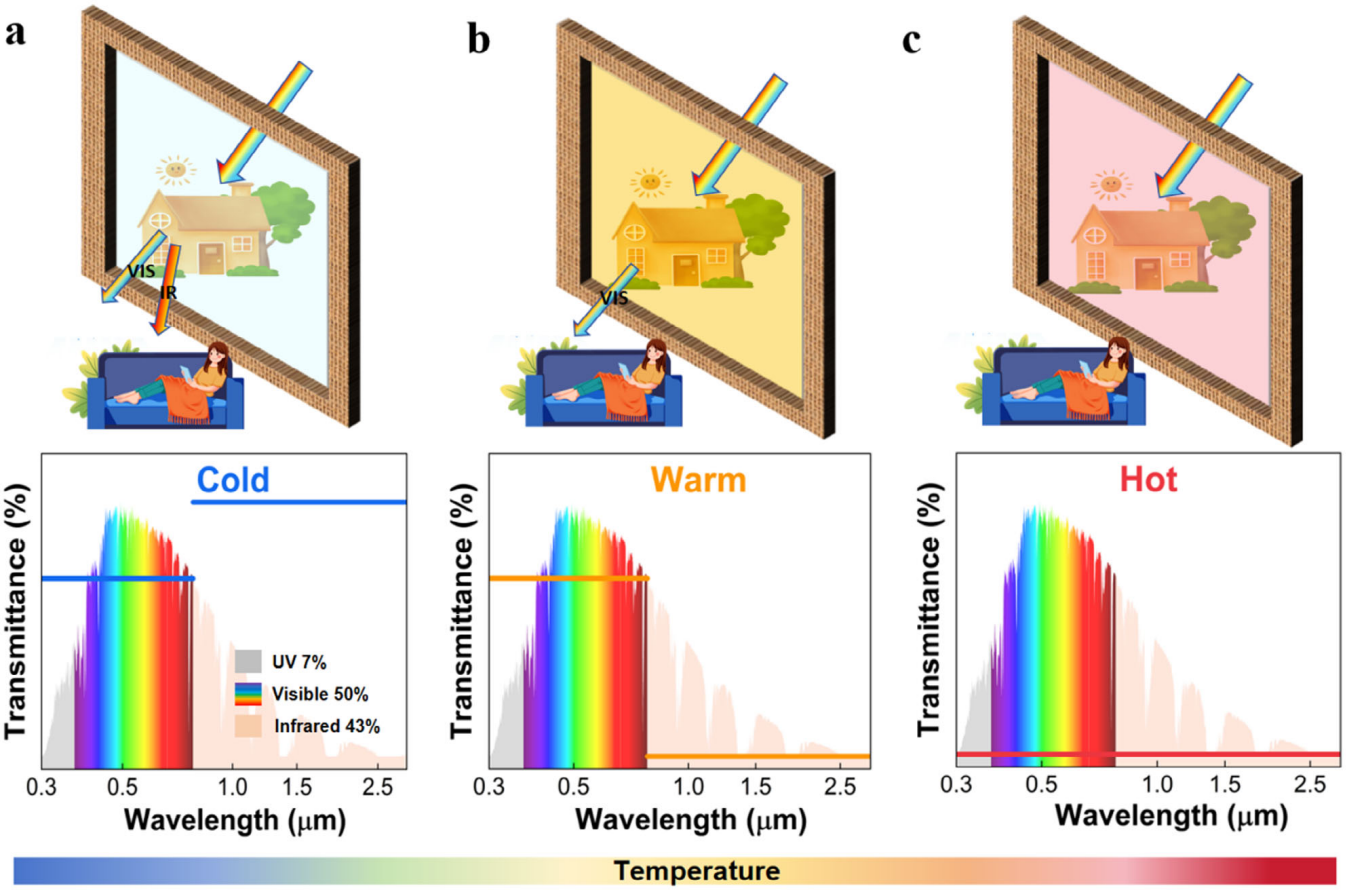
Ideal Three‐State Thermochromic Smart Window. The blue, yellow, and red lines represent the spectra for an ideal energy‐saving smart window in the cold state, warm state, and hot state.

To satisfy human needs in different environments, an ideal smart window with three different states is proposed (**Figure** **1**), which is associated with cold, warm, and hot situations. Specifically, for the cold state, the window maintains a high transmittance in both visible and near‐infrared regions to ensure sufficient transmitted sunlight for lighting and heating. When the temperature rises, the window switches to the warm state, where the window adaptively closes the NIR channel and reduces the solar heat entering the room, but maintains a high visible transmittance to satisfy visual needs. If the temperature continues to rise, the smart window immediately switches to the hot state, blocking most visible and near‐infrared light to reduce building energy consumption. The newly proposed three‐state thermochromic smart window provides broadband tunability of the selective solar spectrum in dynamic climate conditions, promising a significant increase in energy performance on a global scale.

### 2.2 Design Concepts for TSSW Smart Window and W‐VO_2_ Film Characterization

**Figure 2:**
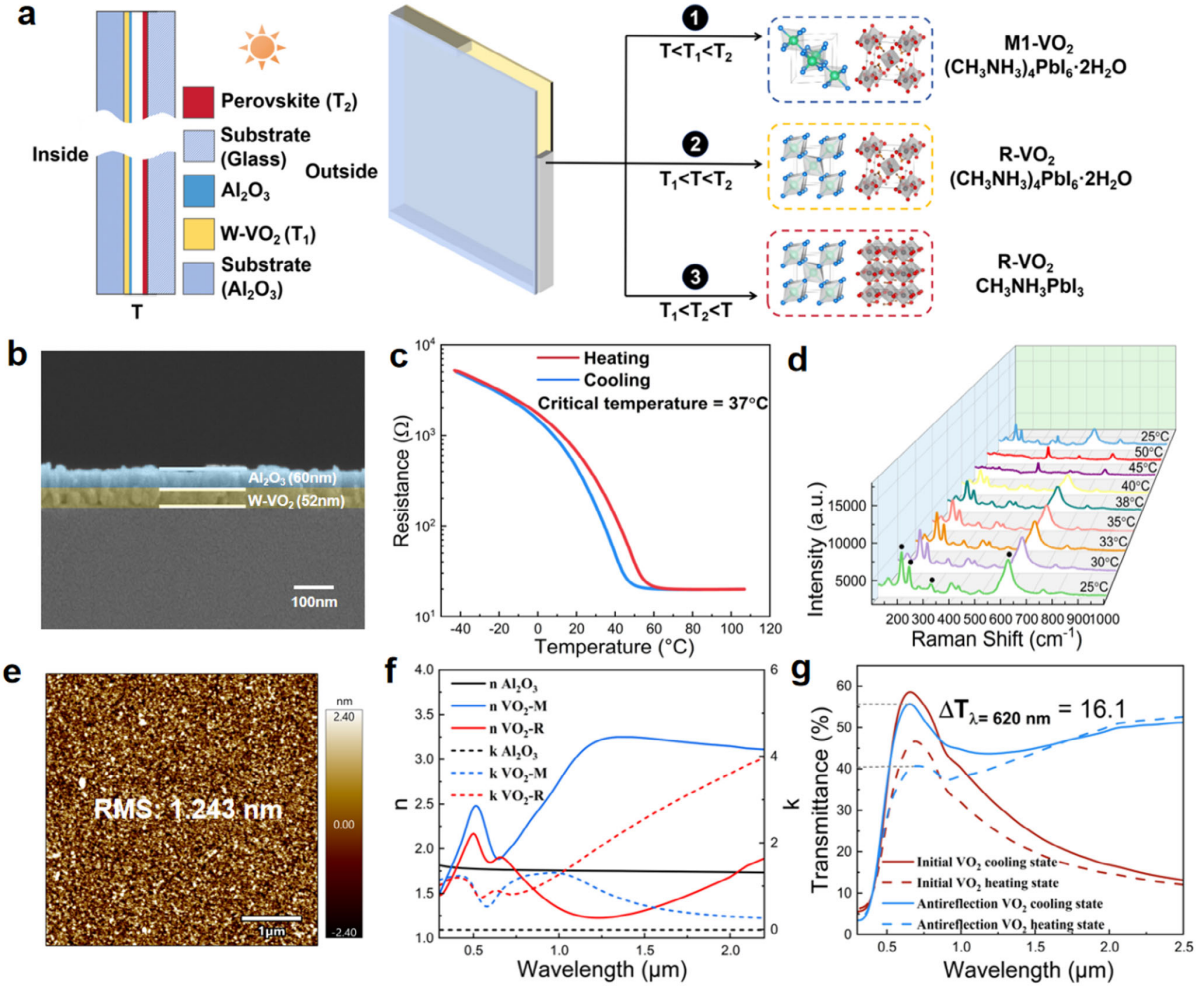
TSSW smart window design principles and related characterization of W‐VO_2_ films. a) Working principle of TSSW window in (1) cold state, (2) warm state, (3) hot state. b) The SEM images for the cross‐section of W‐VO_2_/Al_2_O_3_ films with a sputtering time of 15 min. c) The temperature‐dependent resistance changes for the prepared 52 nm W‐VO_2_ film, showing a critical temperature of ≈37 °C. d) The Raman spectra of W‐VO_2_ film at different temperatures. e) AFM images of W doped VO_2_ thin film, 5 × 5 µm. f) Complex refractive index of VO_2_ in the monoclinic phase (M1) and the rutile phase (R) as well as the Al_2_O_3_ in this study. g) Transmittance spectra of the initial W‐VO_2_ and antireflection W‐VO_2_ in cooling and heating states.

**Figure** **2**a illustrates the design concept of the TSSW smart window, which is composed of an Al₂O₃ substrate, a W‐VO₂ film, an Al₂O₃ film, an air gap, a perovskite film, and a glass substrate, from the left (inside) to the right (outside). The W‐VO₂ film serves to regulate the near‐infrared transmittance, while the perovskite film controls visible light transmittance. It is important to note that the perovskite material's relatively low decomposition energy contributes to its inherent instability. To address this, the functional film is embedded within the window structure, thereby preventing direct exposure to air and enhancing its overall stability. Due to thermal equilibrium through heat conduction, the temperature difference between the inner and outer glass layers is minimal and can be considered as a single temperature, denoted as *T*, and the detailed description is provided in Note S1 (Supporting Information). The critical transition temperatures for the W‐VO₂ and perovskite films are denoted as *T*_1_ and *T*_2_, respectively. By material optimization, the critical temperature of perovskite film is higher than that of W‐VO₂, thus here the temperature *T*_2_ is higher than *T*_1_. Consequently, the TSSW smart window operates in three distinct states based on *T*: 1) **Cold state** (*T* < *T*₁): both the W‐VO₂ and perovskite films remain in their initial phases; 2) **Warm state** (*T*₁ < *T* < *T*₂): the W‐VO₂ film transitions from its monoclinic (M₁) phase to its rutile (R) phase, while the perovskite film remains unchanged; 3) **Hot state** (*T* > *T*₂): the perovskite film also undergoes the structural phase transition.

VO₂ can regulate solar transmittance in the NIR region with temperature change, which is a key material for the preparation of smart windows. Experimentally, we fabricated W‐VO₂ films with varying growth times and measured their resistance‐temperature profiles (Figure S1, Supporting Information) and thicknesses (Figure S2, Supporting Information) to determine the optimal film thickness. As shown in Figure S3 (Supporting Information), visible light transmittance decreased with increased growth time of the W‐VO_2_ films. Considering the modulation capability in the near‐infrared band, we selected a film grown for 15 min, resulting in a thickness of 52 nm (Figure 2b). The critical temperature of the W‐VO_2_ films was maintained at ≈37 °C by adjusting the tungsten doping concentration (Figure 2c). Raman spectroscopy results further demonstrated that the characteristic peaks for monoclinic W‐VO_2_ at 192, 223, 308, 388, and 612 cm^−^¹ disappeared when the heating temperature exceeded 40 °C and reappeared upon cooling to room temperature, confirming the reversible metal‐insulator transition behavior of the W‐VO_2_ films (Figure 2d).^[^ ^23^, ^24^, ^25^ ^]^ The decrease in the phase transition temperature of VO_2_ was attributed to the introduction of excess electrons by the doping of high‐valence W⁶⁺ ions, which reduced the d_II_ band splitting gap and lowered the activation energy required for the transition from the M_1_ phase to the R phase (Figure S4, Supporting Information).^[^ ^22^, ^26^ ^]^ However, the introduction of tungsten atoms led to changes in the grain nucleation and growth process, resulting in a slight decrease in surface roughness (RMS: 1.243 nm) (Figure 2e; Figure S5, Supporting Information).^[^ ^27^, ^28^ ^]^ XPS test results indicated a tungsten doping level of 1.43% (Figure S6, Supporting Information). The high quality of the W‐VO_2_ thin films was further confirmed by X‐ray absorption near edge spectroscopy (Figure S7, Supporting Information).^[^ ^29^ ^]^

To enhance visible wavelength transmittance and increase the weathering resistance of W‐VO_2_ films, an anti‐reflective coating (ARC) was designed and applied. For optimal performance, the ARC layer for W‐VO_2_ films should have a refractive index (n) value in the range of 1.5–2.4 at 550 nm and a thickness of 50–60 nm.^[^ ^30^, ^31^ ^]^ Considering both the fabrication process and coating cost, we selected an alumina film with a thickness of 60 nm, which had a refractive index between that of air and the VO_2_ film (Figure 2f). After introducing the alumina anti‐reflective layer, the optical performance of the multilayer film improved significantly. As shown in Figures 2g and S3 (Supporting Information), the films with an antireflective layer of Al_2_O_3_ showed an increase in transmittance at the peak by more than 15% compared to the single‐layer W‐VO_2_ films. At the same time, the Al_2_O_3_ film further protected the VO_2_ film against oxidation.

### 2.3 Perovskite Film Characterization of TSSW Smart Windows

**Figure 3:**
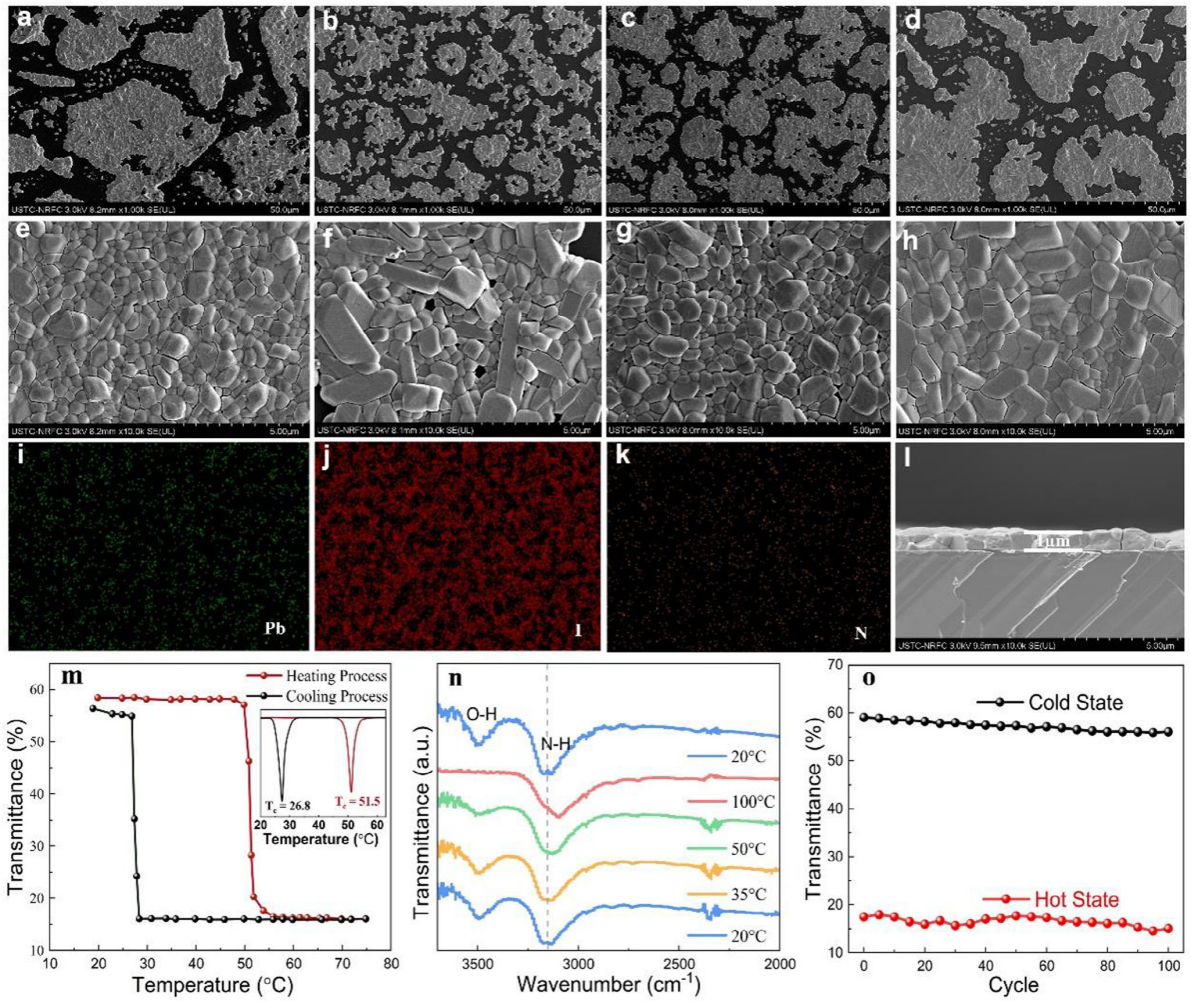
Optimization and characterization of perovskite films. a–d) are the surface scanning electron microscopy (SEM) images (×1 k) of perovskite film with different mixing ratios solvents (DMF: DMSO = 1:0, 4:1, 2:1, 3:2). e‐h) are the surface scanning electron microscopy (SEM) images (×10 k). i‐k) EDS mapping of perovskite film with uniform elemental distribution. l) The SEM images for the cross‐section of perovskite film (DMF: DMSO = 2:1). m) Transmittance of the perovskite film at 550 nm as a function of temperature showing the transition process upon temperature changes. The red arrow indicates the heating process with a temperature increase, while the black arrow indicates the cooling process with a temperature decrease, the transition temperatures for heating and cooling processes are 51.5 and 26.8 °C, respectively. n) FTIR spectrum comparison of thermochromic perovskite at the cold and hot states. o) Cycling performance (*λ = *550 nm) of the thermochromic perovskite film in the ambient environment.

Perovskite thin films are a key part of the preparation of TSSW smart windows. It has been shown that as the CHNH_3_I content in the precursor increases, the reactants do not dissolve completely to synthesize (CH_3_NH_3_)_4_PbI_6_·2H_2_O. No other residues appeared when using a mixture ratio of PbI_2_: CHNH_3_I = 1:4, so this ratio is more suitable for further experiments.^[^ ^32^ ^]^ However, pure DMF is not suitable as a solvent for spin coating alone due to its low viscosity and high volatilization rate. In contrast, DMSO has a slower volatilization rate, and this difference controls the overall volatilization rate of the solvent system, which favors the slow growth of perovskite crystals, and the appropriate amount of DMSO helps to reduce defects and improve film properties.^[^ ^33^, ^34^ ^]^ Therefore, we added different amounts of DMSO to DMF to form mixed solvents (Figure S8, Supporting Information, DMF: DMSO = 1:0, 4:1, 2:1, 3:2). **Figures** **3**a–h showed the surface morphology of the perovskite prepared using different ratios of DMF‐DMSO. When pure DMF solvent was used, more holes and poor coverage of the film surface were observed in Figures 3a,e. When the percentage of DMSO was increased to 25 vol%, the holes on the surface of the films were reduced as shown in Figures 3b,f. The surface of the films was covered with holes. Continuing to increase the DMSO percentage to 33 vol%, it was found that the grain size of the film surface became smaller and the coverage further improved (Figures 3c,g). However, as the percentage of DMSO was increased to 40 vol%, the coverage of the film surface showed a decreasing trend (Figures 3d,h), so 33 vol% DMSO added to DMF was finally selected as the optimal solvent ratio.

The uniform distribution of elements on the surface of the perovskite film with a thickness of ≈1 µm can also be demonstrated by SEM mapping tests in Figures 3i–k and cross‐sectional characterization in Figure 3l. The transmission spectra of the chalcogenide film are illustrated in Figure S10 (Supporting Information). The τ_lum_ is 77.4% and 43.4% in the cold and hot states, respectively. The transmittance and hysteresis lines (λ = 550 nm) of the perovskite coatings were collected at variable temperatures at 2 °C intervals in Figure 3m. This phenomenon was attributed to the different energy barriers between the heating and cooling processes (dehydration and hydration processes). The transmittance at 550 nm was chosen to monitor the thermochromic effect because the maximum difference in perovskite transmittance was observed at 550 nm,^[^ ^32^, ^35^ ^]^ which also corresponded to the peak of the CIE brightfield luminescence efficiency of the human eye. From the transmittance spectral the average critical temperature was 51.5 °C. Figure 3n shows the FTIR spectra of thermochromic perovskite in the hot and cold states. From the spectra, when the sample was at 20 °C, a distinct characteristic peak of O─H bonding appeared near 3500 cm^−1^. When the sample was heated to 100 °C, the O─H bond completely disappeared and the N─H chemical bond moved to the low wave number direction. When the sample was cooled to 20 °C, the O─H bond reappeared and the N─H chemical bond returned to its original position, indicating that water molecules play a key role in the weakening of the hydrogen bond strength.^[^ ^36^ ^]^

In addition, cycling performance was investigated to ensure its suitability for TSSW smart window applications. To verify its stability during heating and cooling, 100 cooling and heating cycles were performed. The ambient temperature was set at a constant 25 °C with an RH of ≈50% and the heating and cooling temperatures during the cycling tests were set at 80 and 25 °C, respectively. During the heating and cooling cycles, the transparent state (i.e., cold state) and the reddish‐brown state (i.e., hot state) were consistently displayed throughout the cycling tests (Figure S11 and Movie S1, Supporting Information). The transmittance of perovskite at 550 nm in both the hot and cold states was examined by testing the transmittance at five‐cycle intervals, and the results showed that the transmittance did not decay significantly after many heating and cooling cycles, demonstrating their stable and promising thermochromic properties.

### 2.4 Application of the TSSW Smart Window in the Real Environment

**Figure 4:**
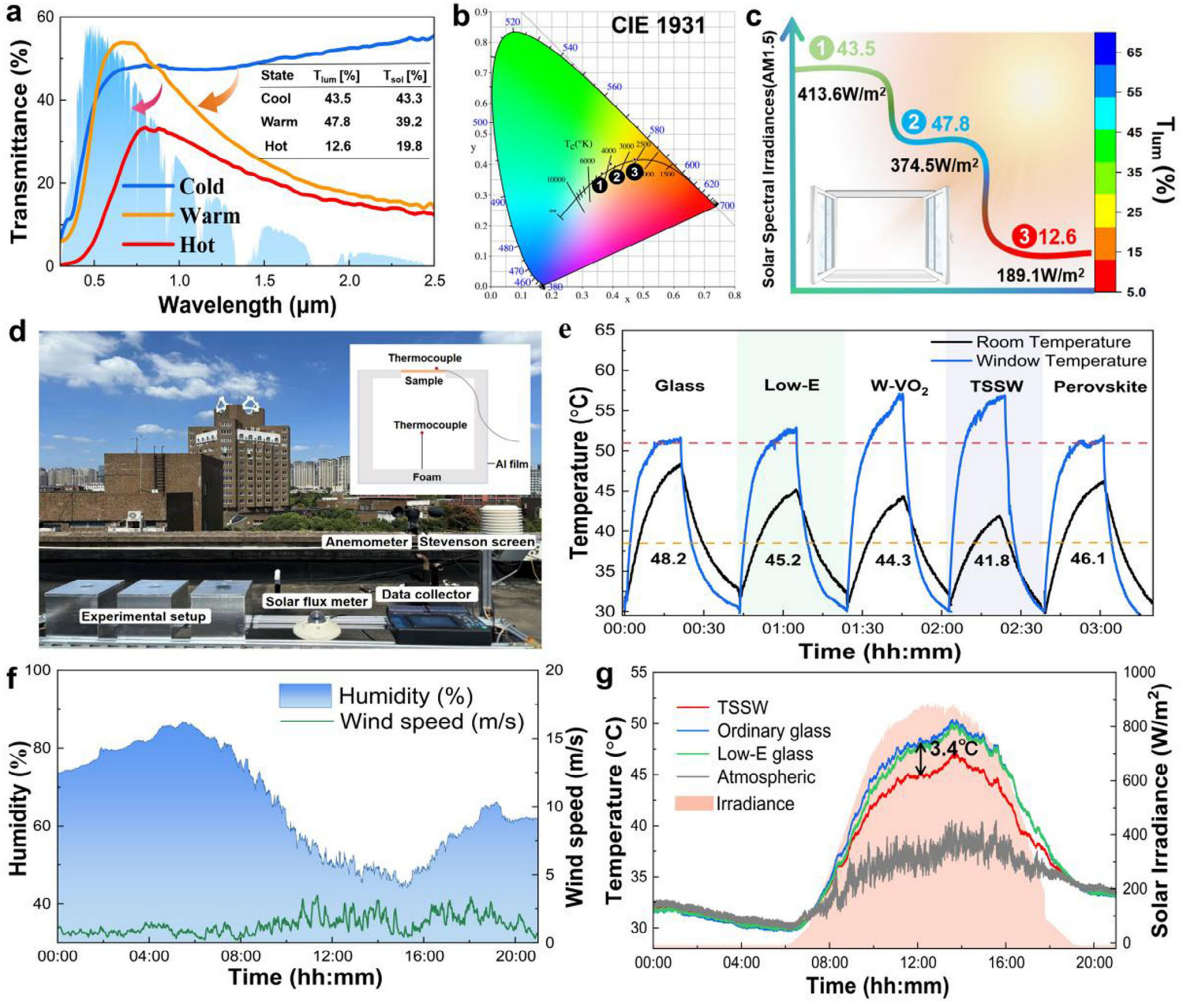
Spectral Properties and Outdoor Performance of TSSW. a) Transmittance spectrum (0.35–2.5 µm) of the TSSW smart window at cool (25 °C), warm (45 °C), and hot (60 °C) states, AM 1.5 G, air mass 1.5 global. b) CIE chromaticity coordinate chart for TSSW smart window Three‐State color change. The smart window color gradually changes from light yellow to orange. c) The visible transmittance and the amount of solar irradiation entering the room correspond to the TSSW smart window. d) Scheme of model house field test set up. e) Temperature profiles for different types of windows, sun simulator exposure time 20 min, stop 20 min, irradiation intensity fixed at 970.8 W/m^2^. f) Wind speed and humidity on the day of the outdoor experiment. g) Room temperature curve for the model house field test in Hefei.

As shown in **Figure** **4**a, the designed adaptive dual‐band TSSW smart window can independently regulate visible and NIR light, enabling the transition between cold, warm, and hot states. This smart window achieves a maximum visible light transmittance of 47.8% and a solar modulation efficiency of 23.5%. Notably, in the warm state, the visible light transmittance remains unaffected by the NIR light shielding, ensuring the visual needs of humans are met. In the hot state, the visible light and solar transmittance decrease to 12.6% and 19.8%, respectively. In contrast to current work related to thermochromic smart windows, the TSSW smart window not only achieves a three‐state switching function that improves the ability to modulate the visible and solar spectrum. In addition, potential leakage problems with materials such as liquid paraffin are avoided (Table S1, Supporting Information). In window applications, human color perception is crucial for evaluating overall color rendering performance. Based on the transmission spectrum of the TSSW smart window, the color coordinates in the CIE 1931 chromaticity diagram were calculated, as shown in Figure 4b. As the state switches, the color gradually shifts from light yellow to orange‐red. In addition, visible light transmittance and total indoor irradiance in different states were calculated and correlation curves were plotted (Figure 4c) to evaluate the impact of TSSW smart windows on human visual and thermal perception. The TSSW smart window blocked NIR light while maintaining a high visible light transmittance to ensure a good visual experience. With the conversion of the smart window's working state, the total solar irradiance entering the room was significantly reduced from 413.6 W/m^2^ in hot state to 189.1 W/m^2^ in cold state, with a modulation ratio as high as 54.3%, which was in line with the energy saving requirements of green buildings. Immediately following this, the feasibility of the TSSW three‐state switch was initially verified using a solar simulator, where the phase transition occurs earlier than in the perovskite film due to the initial absorptivity of the W‐VO_2_ film to ensure that a stepwise and reasonable modulation of the solar spectrum is achieved (Figure S13, Supporting Information). To further verify the superiority of TSSW smart windows, we designed five different types of smart windows and conducted indoor experiments. For more information about these five types of smart windows, please refer to Figure S15 (Supporting Information). As shown in Figure 4d, we recorded five temperature profiles for each of the chamber and the outer surface of the window, respectively. As expected, the chamber temperature of the TSSW smart window design was significantly lower than the other chamber designs, especially when compared to the glass chamber, with a temperature difference of 6.4 °C after only 20 min of irradiation.

Subsequently, to test the practicality of the TSSW smart window in real outdoor environments, we conducted comparative experiments with commonly used double‐glazed glass in the market (e.g., Low‐E glass and ordinary glass). The test was conducted in Hefei in early autumn (see Table S2, Supporting Information for weather information), and the samples were installed on an insulated acrylic model house with a volume of 16 × 16 × 10 cm^3^. The experimental setup is shown in Figure 4e, and the tests were conducted on the roof of the building. The windows of the model house face the sky to simulate roof windows. We used two T‐type thermocouples to monitor the indoor air temperature and the surface temperature outside the window, respectively, and measured humidity, wind speed, and total solar radiation via a weather station. The test results are shown in Figures 4f,g, and S16 (Supporting Information). After sunrise (6:00 AM), the indoor air temperatures of the three sample houses increased rapidly as the intensity of solar radiation increased. During the day, the indoor air temperature of the model house with TSSW smart windows was consistently lower than that of the other model houses, even though the outside surface temperature of TSSW windows was the highest, which confirmed the shielding effect of TSSW against solar radiation. The maximum indoor air temperature reduction was 3.4 °C at noon. It was noteworthy that the difference in indoor air temperatures between the model house with Low‐E smart windows and the normal glass house was small and almost non‐existent in the afternoon. This suggested that the indoor air temperature of each model house continued to rise under continuous solar radiation, while the Low‐E smart window had little cooling effect due to its low emissivity on the inner surface, which made it difficult for heat to be transferred to the outdoors by radiation. When the intensity of solar radiation decreases in the late afternoon, the indoor air temperature difference between the three model houses gradually decreases, which suggests that the TSSW smart window controls energy loss or gain mainly by regulating solar radiation. Overall, the field test results showed that the TSSW smart window could effectively regulate solar transmittance and demonstrated great potential as a smart window in building applications.

The optical stability of TSSW smart windows is critical to their practical application. Tests have shown that under UV exposure and moderate humidity (50% RH), TSSW smart window maintain stable optical and thermal properties and exhibit superior durability. While high humidity (80% RH) has an impact on performance, this is rare in natural environments and is of short duration. Therefore, these results confirm the practical feasibility and certain use of the TSSW smart window (Figures S17 and S18, Supporting Information).

### 2.5 Energy Efficiency of Buildings Integrated with the Smart Window

**Figure 5:**
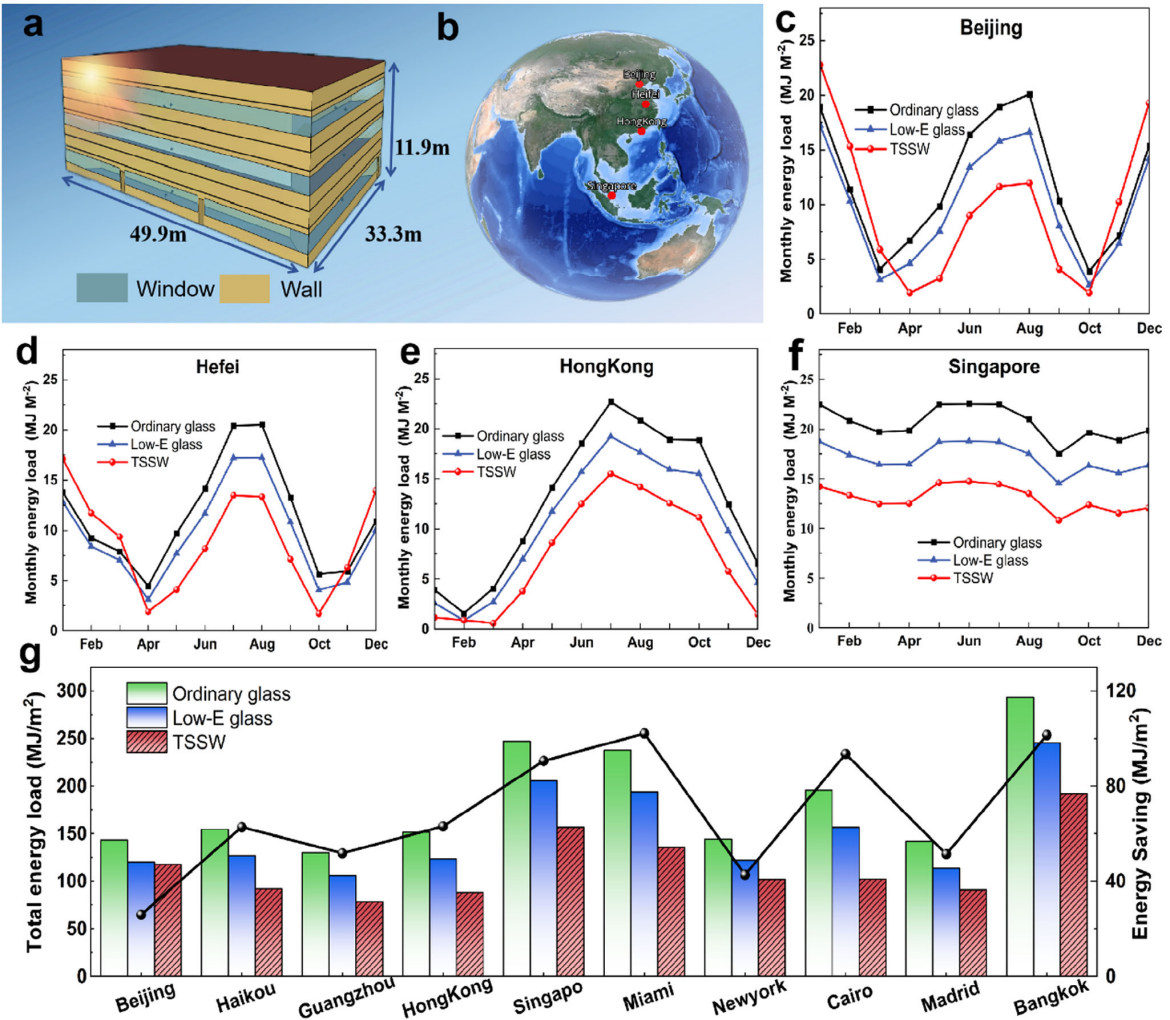
Simulation for energy‐saving performance in buildings. a) Building energy consumption calculation model, 49.9 m (length) * 33.3 m (wide) * 11. 9 m (high). b) The geographic location of Beijing, Hefei, Hong Kong, and Singapore. c–f) Monthly HAVC energy consumption of Ordinary glass, Low‐E windows, and TSSW smart windows in Beijing, Hefei, Hong Kong, and Singapore. g) Annual HAVC energy savings performance for Ordinary glass, Low‐E windows, and TSSW smart windows in each city climate.

To further evaluate the energy‐saving performance of smart windows in real buildings, an Energy Plus‐based energy estimation model was developed, and a 3‐floor reference office building constructed by the U.S. Department of Energy (DOE) was used in the simulation (**Figure** **5**a). To investigate the applicability of TSSW smart windows in different climate zones, four cities with different latitudes, namely Beijing, Hefei, Hong Kong, China, and Singapore, were selected (Figure 5b). Unsurprisingly, the energy‐saving ability of TSSW smart windows was significantly better than that of ordinary glass and Low‐E glass. Among them, the total consumption in Hong Kong (subtropical) and Singapore (tropical) was lower than that of ordinary glass and Low‐E glass for 12 months of the year (Figures 5c–f). The energy savings of TSSW smart windows were particularly significant in subtropical and tropical regions, as the original huge amount of energy used by building cooling systems and auxiliary equipment can be significantly reduced. Adopting TSSW window technology in these regions could contribute even more to building carbon neutrality. Furthermore, we comprehensively analyzed the total annual energy demand with TSSW smart windows across 10 different latitudes globally (as illustrated in Figure 5g and Table S3, Supporting Information). The regions that stand out in terms of the most significant reduction in total annual energy demand are Miami, Bangkok, and Singapore, with respective decreases of 42.97%, 34.59%, and 36.59% compared to regular glass. The Miami region demonstrated the potential to save up to 102.09 WJ/m^2^ in total annual energy demand. The cumulative effect of TSSW smart windows on building energy savings is significant from a long‐term operational perspective.

## 3 Conclusion

In this study, we have designed a three‐state thermochromic smart window with W‐VO_2_/perovskite films that demonstrates dynamic and independent regulation of NIR and visible light transmittance, thereby adaptively categorizing the smart window into cool, warm, and hot states. Its double glazing further enhances environmental resilience and durability. The smart window exhibits reversible color changes, with transmittance values of τ_lum_ = 47.8% in warm states, 12.6% in hot states, and a solar modulation capacity of Δτ_sol_ = 23.5%. Most importantly, in field tests using the model house equipped with the TSSW, the indoor air temperature was reduced by 3.4 °C, showing great potential for energy savings compared to conventional windows. The energy savings of the device in different climate zones are remarkable. This work provides a viable avenue for year‐round thermal management, which is promising for energy‐saving applications in the future.

## 4 Experimental Section

### Materials

CH_3_NH_3_I (MAI, 99.5%) and PbI_2_ (99%) were provided by Xi'an Polymer Light Technology. Dimethylformamide (DMF, ≥ 99.5%) and Dimethyl sulfoxide (DMSO, ≥ 99.5%) were purchased from Alfa Assar. W target (> 99.99%) and V target (> 99.99%) were purchased from Zhongnuo New Material Technology Co. Al_2_O_3_ substrates were purchased by Shanghai Spectrum Power Precision Instrument Technology Co (PrMat).

### Preparation of TSSW

W‐VO_2_ films with different thicknesses were prepared by wet oxidation method with the sputtering times of 10, 15, and 20 min for sputtering V, and 1 min 10 s, 1 min 25 s, and 1 min 35 s for sputtering W to ensure that the critical temperature of W‐VO_2_ films was maintained at ≈35–40 °C. The growth process was as follows: first, W‐doped V metal film was deposited on the substrate (Al_2_O_3_) by RF magnetron co‐sputtering. The RF sputtering powers of the metal W target and V target used were 25 W and 150 W, respectively, and the working pressure was maintained at 1.0 Pa with an argon flow rate of 80 SCCM. The precursor films were then oxidized to W‐VO_2_ films by wet oxidation in a tube furnace. Finally, 60 nm thick Al_2_O_3_ was deposited on the top surface of the obtained films.

A one‐step method was used to fabricate the perovskite film. An amount of PbI_2_ and CH_3_NH_3_I was dissolved in DMF and DMSO solvents and stirred vigorously in a water bath at 60 °C for 24 h. The exact amount of chemicals for the perovskite precursor with different mass ratio ingredients could be found in Table S4 (Supporting Information). Glass of 1 mm thickness was first cleaned in an ultrasonic bath with acetone, ethanol, and deionized water for 10 min and then dried with N_2_. They were then treated in a 200 W plasma cleaner (PLUTO‐30) for 20 min with oxygen gas. Perovskite film was spin‐coated on the treated surface of the substrates, with a certain amount of the precursor, 500 rpm for 10 s and a high rate of 3000 rpm for 35 s. The samples were then dried on a hot plate at 100 °C for 30 min to remove excess solvent.

Subsequently, two substrates (two inches) were sealed together to form a double‐glazed window, ensuring that the film‐bearing side was insulated from outside air and further protecting the films.

### Characterization of TSSW

The electric property of the W‐VO_2_ was measured by a physical property measurement system (PPMS) using the Vander Pauw configuration under temperatures varying from −50 to 120 °C with a sweeping rate of ≈1 °C/min. An integrated laser Raman system (LabRAM HR; 523 nm laser source) recorded the temperature‐dependent Raman spectra with a laser power of 5 mW. The surface morphologies and roughness of W‐VO_2_ film were examined by atomic force microscopic (AFM, MFP‐3D‐Origin). Scanning Electron Microscopy (SEM, FESEM SU8220, Hitachi) measured the surface morphologies and the cross‐section images of the films. X‐ray photoelectron spectroscopy (XPS) (Kratos, AXIS Supra+, Al Kα X‐ray of 1486.6 eV) was used to characterize the valence state of V. The X‐ray absorption near‐edge spectroscopy (XANES) was conducted at the XMCD beamline (BL12B) in the National Synchrotron Radiation Laboratory (NSRL), Hefei, to further characterize the chemical state of the films. The crystal structure was characterized by XRD (Multifunctional Rotating‐anode X‐ray Diffractometer, Rigaku SmartLab(9)). The UV durability test was conducted using an LED UV lamp (50 W, 365 nm) to simulate prolonged UV exposure. Humidity cycling tests were conducted in a constant temperature and humidity chamber at 30 °C with relative humidity of 50% and 80% to assess stability under different humidity conditions.

### Optical Measurements

The spectral transmittance and reflectance of the smart window within the solar radiation band were measured using the UV–vis‐NIR spectrometer (SolidSpec‐3700DUV, Shimadzu) equipped with an integrating sphere detector. A temperature controller was paired to regulate the temperature of the sample to measure in both the cold and hot states. To quantify the amount of solar thermal energy entering a building via solar transmittance, visible transmittance *τ*_lum_ is defined in Equation (1), solar transmittance τ_sol_ is defined in Equation (2):

(1) $$\tau_{lum}=\frac{\int_{\lambda \;=\;380\;\mathrm{nm}}^{780\;\mathrm{nm}}\bar{y}\left(\lambda \right)\tau \left(\lambda \right)d\lambda }{\int_{\lambda \;=\;380\;\mathrm{nm}}^{780\;\mathrm{nm}}\bar{y}\left(\lambda \right)d\lambda }$$

(2) $$\tau_{sol}=\frac{\int_{\lambda \;=\;300\;\mathrm{nm}}^{2500\;\mathrm{nm}}{AM}_{1.5}\left(\lambda \right)\tau \left(\lambda \right)d\lambda }{\int_{\lambda \;=\;300\;\mathrm{nm}}^{2500\;\mathrm{nm}}{AM}_{1.5}\left(\lambda \right)d\lambda }$$

where *τ*(λ) is the transmittance of the smart windows at wavelength λ. The CIE (International Commission on Illumination) standards for photopic luminous efficiency of the human eye ($\bar{y}$(λ)), and the solar irradiance spectrum for an air mass of 1.5 (AM_1.5_(λ)) were used as weighting functions for the wavelength‐dependent transmittance.^[^ ^9^, ^37^, ^38^ ^]^

The thermal reflectivity within the mid‐infrared wavelength band was performed using an FTIR spectrometer (Nicolet iS50) equipped with an integrating sphere (PIKE). All reflectivity spectrums were normalized using a standard gold film.^[^ ^39^ ^]^

(3) $$R_{\mathrm{MIR}}=\frac{\int R(\lambda )B(\lambda )d\lambda }{\int B(\lambda )d\lambda }$$

### Outdoor Experiment Measurements

The outdoor experiments were conducted on the rooftop of the second building of Mechanics at the University of Science and Technology of China, Hefei (31°50′24′N, 117°15′1″E). Total solar radiation was measured by a pyranometer (TBQ‐2, Jinzhou Sunshine Technology Co., Ltd) that was installed in parallel with samples with an uncertainty of ± 2%. An integrated weather station (HSTL‐BYXWS) was applied to measure the ambient temperature and relative humidity. T‐type thermocouples with an accuracy of ± 0.5 °C were used for the temperature of the setup. The temperature and humidity data were recorded using a data acquisition instrument (LR8450, HIOKI).

### Energy Saving Performance Simulation

To evaluate the effect of smart windows on the heating/cooling load of buildings, we develop a single‐story small office building for energy‐saving simulation. The dimensions of the building are 49.9 m × 33.3 m × 11.9 m, with windows size of 2 m × 8 m evenly distributed on all four walls (Figure 5a), with a window‐to‐wall ratio of 33.4%. The building data were summarized in Table S5 (Supporting Information) in the Supporting Information, and the optical properties of the ordinary glass, Low‐E glass, and TSSW smart window used in the simulation are given in Table S6 (Supporting Information) in the Supporting Information. The spectral data for the smart window was generated based on actual measurements, while the spectral data for common glass was from existing literature. The thermochromic function in Energy Plus was utilized to simulate the thermal chromic characteristics of the smart window. The cooling temperature of the room in the summer cooling season was set at 26 °C, and the heating temperature in the winter heating season was set at 20 °C. To approximate the real situation, the temperature for the first switching state was set to 37 °C and the temperature for the second switching state was set to 52 °C. The energy efficiency of the building was demonstrated by comparing the heating/cooling load under smart windows and conventional glass cases.

## Conflict of Interest

The authors declare no conflict of interest.

## Author Contributions

M.‐L.L. designed the experiments and wrote the manuscript. X.‐S.L. and W.‐S.Z. provided experimental support. L.‐X.L. analyzed the data. L.L. and C.‐M.W. gave suggestions for the research. G.P., B.Z., and C.‐W.Z. revised the manuscript, supervised the work, and provided funding.

## Supporting information



Supporting Information

Supplemental Movie 1

## Data Availability

The data supporting the plots within this paper and other study findings are available from the corresponding author upon reasonable request.
