# Supplementary material for: Three‐State Thermochromic Smart Window for Building Energy‐Saving
Source: Adv Sci (Weinh). 2025 Mar 20;12(18):2416688. doi: 10.1002/advs.202416688 (PMC12079551; doi:10.1002/advs.202416688)
Supplement: Supplementary file 1 — Supporting Information [file ADVS-12-2416688-s001.docx]

**Supplementary Information for**

**Three-State Thermochromic Smart Window for Building Energy-Saving**

*Meiling Liu^1+^, Xiansheng Li^2^, Wenshuo Zhang^2^, Lanxin Li^2^, Liang Li^1^, Chengming Wang^3^, Gang Pei^2*^,* *Bin Zhao^2*^, Chongwen Zou^1*^*

^1^ National Synchrotron Radiation Laboratory, School of Nuclear Science and Technology, University of Science and Technology of China, Hefei, Anhui 230029, P. R. China

^2^ Department of Thermal Science and Energy Engineering, University of Science and Technology of China, Hefei, 230027, P.R. China

^3^Instruments' Center for Physical Science, Hefei National Research Center for Physical Sciences at the Microscale, University of Science and Technology of China

*Corresponding Author: zb630@ustc.edu.cn

*Corresponding Author: peigang@ustc.edu.cn

*Corresponding Author: czou@ustc.edu.c

This PDF file includes:

Supplementary Figs. 1 to 18

Supplementary Table 1 to 7

**Supplementary Note 1**

**Description of TSSW Smart Window** **a Single Temperature (T)**

As the outdoor experiment cannot simulate the use of smart windows in real situations, resulting in a certain temperature difference between the inner and outer glass layers of the TSSW smart window, there is a deviation from the assumption in section 2.2. However, even with this temperature differential, the three-state switching functionality of the TSSW can still be achieved.

Here we explain further: the critical temperature of W-VO_2_ is *T_1_*, the critical temperature of perovskite is *T_2_* (*T_1_<T_2_*, consistent with the manuscript), and the temperature of the W-VO_2_ surface in the test is *T_v_,* the temperature of the perovskite surface is *T_p_*, as shown in supplementary Figure S14.

Due to the intrinsic solar absorption of W-VO_2_ film, the surface temperature *T_v_* is higher than the temperature of perovskite film *T_p_* (*T_v_* > *T_p_*) during the testing process, while the critical temperature of the design of the TSSW presents the relationship of *T_1_<T_2_*, which inevitably leads to the phase transition process of the W-VO_2_ film is earlier than that of the perovskite film, and is also the prerequisite for the realization of the three-state switching.

For simplification purposes in the theoretical analysis, the overall temperature of the TSSW is still assumed to be a uniform *T* in the manuscript.

**Table:**

**Table S1**. Optical and transition properties of thermochromic smart windows.

| **Materials** | **T_lum_ [%]** | **T_sol_ [%]** | **T_C_ [°C]** | **States** | **Year** | **Work** |
| --- | --- | --- | --- | --- | --- | --- |
| PNIPAm^[1]^ | 28 | 20.4 | 32 | Hot→Cold | 2014 | 1 |
| PNIPAm/CsxWO_3_^[2]^ | 10.4 | - | 30 | Hot→Cold | 2018 | 2 |
| HPC^[3]^ | 37.5 | 25.7 | 38 | Hot→Cold | 2016 | 3 |
| H-MAPbI_3-x_Cl_x_^[4]^ | 54.9 | 23.7 | 40.1 | Hot→Cold | 2021 | 4 |
| VO_2_^[5]^ | - | 8.8 | 43.4 | Hot→Warm | 2013 | 5 |
| VO_2_/SiO_2_^[6]^ | 1.7 | 7.1 | 68 | Hot→Warm | 2014 | 6 |
| PMMA/VO_2_^[7]^ | 7.3 | 17 | 30 | Hot→Cold | 2021 | 7 |
| PNIPAm/AgNW^[8]^ | 57.1 | 58.4 | 31 | Hot→Cold | 2022 | 8 |
| W_x_V_1-x_O_2_/paraffin/PVA^[9]^ | 36.5 | 33.7 | 32.9/58 | Hot→Warm→Cold | 2024 | 9 |
| **This work** | 30.9 | 23.5 | 37/51.5 | Hot→Warm→Cold | - | - |

**Table S2.** The weather status of Heifei on Aug. 24th, 2024 measured by the weather station.

Date Minimum Air Temperature(°C) Maximum Air Temperature(°C) Relative Humidity

Aug. 24th 29.9 40.5 65%

**Table S3**. Climate information of cities used in the simulation.

| City | Location | Climate Types | Mean Temperature |
| --- | --- | --- | --- |
| **Beijing** | 39°56′N, 116°20′E | Temperate monsoon | 9.0 °C – 19.0 °C |
| **Heifei** | 30°57′N, 116°41′E | Subtropical monsoon | 11.0 °C – 21.0 °C |
| **HongKong** | 22°08′N, 113°49′E | Subtropical monsoon | 17.0 °C – 29.0 °C |
| **Singapore** | 1°18′N, 103°51′E | Tropical rainforest | 26.5 °C -28.5 °C |
| **Haikou** | 19°32′N, 110°10′E | Temperate marine | 23.0 °C -29.0 °C |
| **Guangzhou** | 22°26′N, 112°57′E | Subtropical monsoon | 21.5 °C -22.2 °C |
| **Miami** | 25°46′N, 80°12′W | Temperate monsoon | 20.0 °C -28.0 °C |
| **Newyork** | 40°42′N, 74°0′W | Subtropical monsoon | 8.0 °C -15.0 °C |
| **Cairo** | 30°03′N, 31°15′E | Mediterranean climate | 18.0 °C -31.0 °C |
| **Madrid** | 40°25′N, 3°45′W | Temperate continental | 9.0 °C -19.0 °C |
| **Bangkok** | 13°45′N, 100°31′E | Tropical monsoon | 26.0 °C -34.0 °C |

**Table S4.** The exact amount of chemicals used in experiments.

Precursors PbI_2_ (g) CH_3_NH_3_I (g) DMF (ml) DMSO (ml) DMF: DMSO

2.00

0.50

4:1

1:0

0.00

2.50

1.1128

1.1128

0.807

1

1.67

0.83

2:1

1.1128

0.807

2

3:2

0.807

3

1.00

1.50

1.1128

0.807

4

**Table S5.** Thermophysical parameters of the baseline building envelope.

| Construction | Material  (outside to inside) | Thickness  (mm) | Conductivity  (W/(m·K)) | Density  (kg/m3) | Specific heat  (J/(kg·K)) |
| --- | --- | --- | --- | --- | --- |
| Exterior wall | Stucco | 25.4 | 0.72 | 1856 | 840 |
|  | Gypsum board | 15.9 | 0.16 | 800 | 1090 |
|  | Wall insulation (no mass) | Thermal resistance is 2.81828 m^2^·K/W. | | | |
|  | Gypsum board | 15.9 | 0.16 | 800 | 1090 |
| Interior wall | Gypsum board | 25.4 | 0.16 | 800 | 1090 |
| Floor 1 | Concrete | 203.2 | 2.31 | 2322 | 832 |
|  | Carpet pad (no mass) | Thermal resistance is 0.21648 m^2^·K/W. | | | |
| Floor 2 | Concrete | 101.6 | 2.31 | 2322 | 832 |
|  | Carpet pad (no mass) | Thermal resistance is 0.21648 m^2^·K/W. | | | |
| Ceiling | AC02 Acoustic Ceiling | 12.7 | 0.057 | 288 | 1339 |
| Roof | Built-up roofing | 9.5 | 0.16 | 1120 | 1460 |
|  | Roof insulation (no mass) | Thermal resistance is 5.30668 m^2^·K/W. | | | |
|  | Metal surface | 0.8 | 45.28 | 7824 | 500 |
| Door | Opaque panel | Thermal resistance is 0.47596 m^2^·K/W. | | | |

**Table S6**. Optical information of the windows used in the simulation.

|  | Ordinary glass | Low-E Window | TSSW smart window | | |
| --- | --- | --- | --- | --- | --- |
| **States** | - | - | Cold | Warm | Hot |
| **Solar Transmittance** | 0.837 | 0.630 | 0.433 | 0.392 | 0.198 |
| **Solar Front Reflectance** | 0.075 | 0.190 | 0.362 | 0.332 | 0.327 |
| **Solar Back Reflectance** | 0.075 | 0.220 | 0.251 | 0.296 | 0.358 |
| **Visible Transmittance** | 0.898 | 0.850 | 0.435 | 0.478 | 0.126 |
| **Visible Front Reflectance** | 0.081 | 0.056 | 0.215 | 0.181 | 0.161 |
| **Visible Back Reflectance** | 0.081 | 0.274 | 0.309 | 0.296 | 0.079 |
| **Emissivity (Front side)** | 0.840 | 0.100 | 0.752 | 0.752 | 0.752 |
| **Emissivity (Back side)** | 0.840 | 0.900 | 0.933 | 0.933 | 0.933 |

**Table S7.** Optical properties of smart windows in three states.

State T_lum_ [%] T_sol_ [%]

1 43.5 43.3

2  47.8 39.2

3 12.6 19.8

**Figures:**


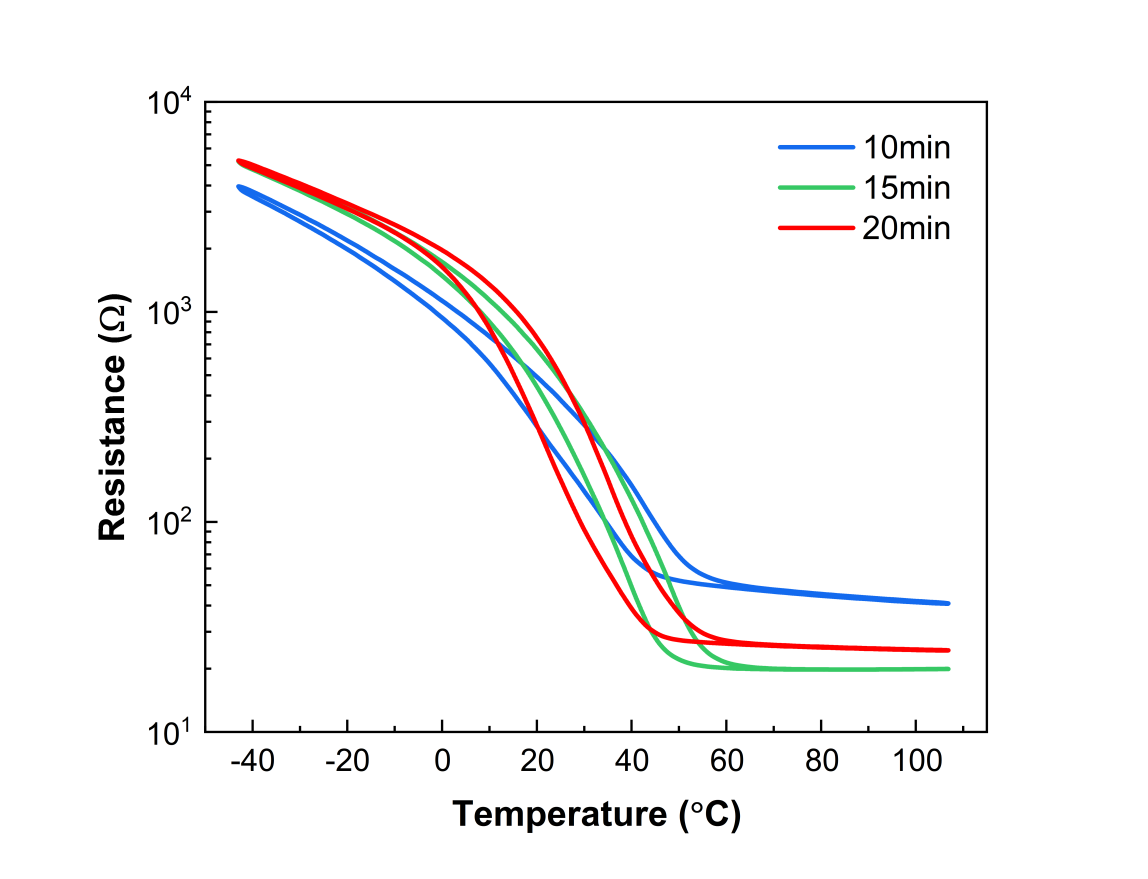


**Figure. S1** The temperature-dependent resistance of the prepared W-VO_2_ thin films with different growth times varied with the critical temperature of about 37℃.


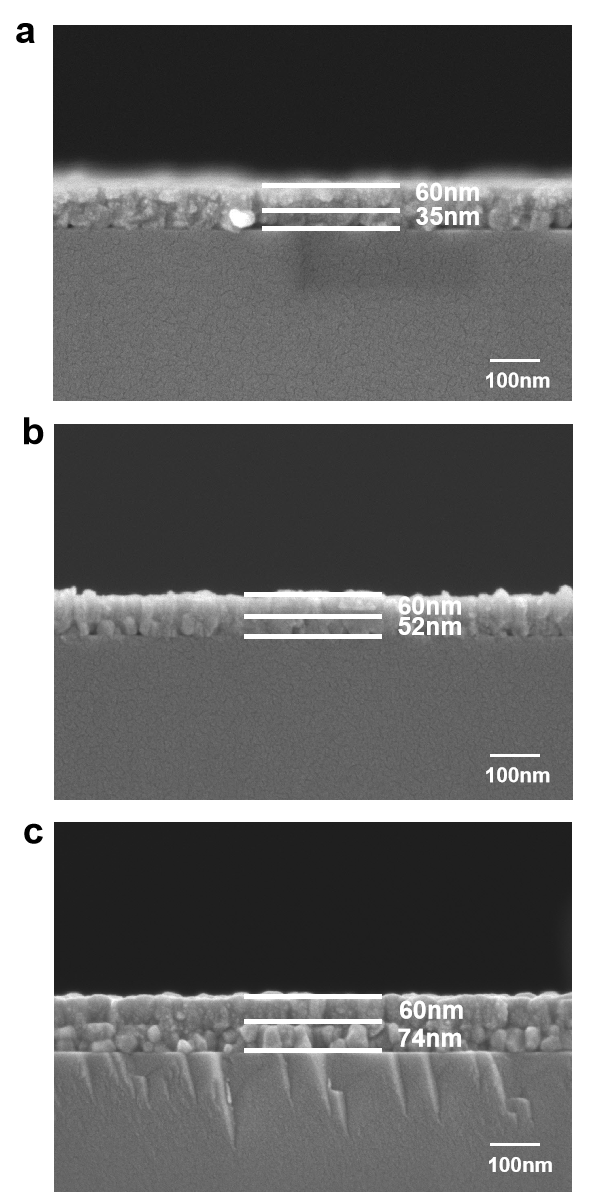


**Figure. S2** SEM images of W-VO_2_ film cross sections grown for 10 min, 15 min, and 20 min. **a)** The thickness of W-VO_2_ was 35 nm for 10 min，**b)** 52 nm for 15 min, **c)** and 74 nm for 20 min. The topmost layer is 60 nm Al_2_O_3_ film, which acts as an anti-reflective layer.


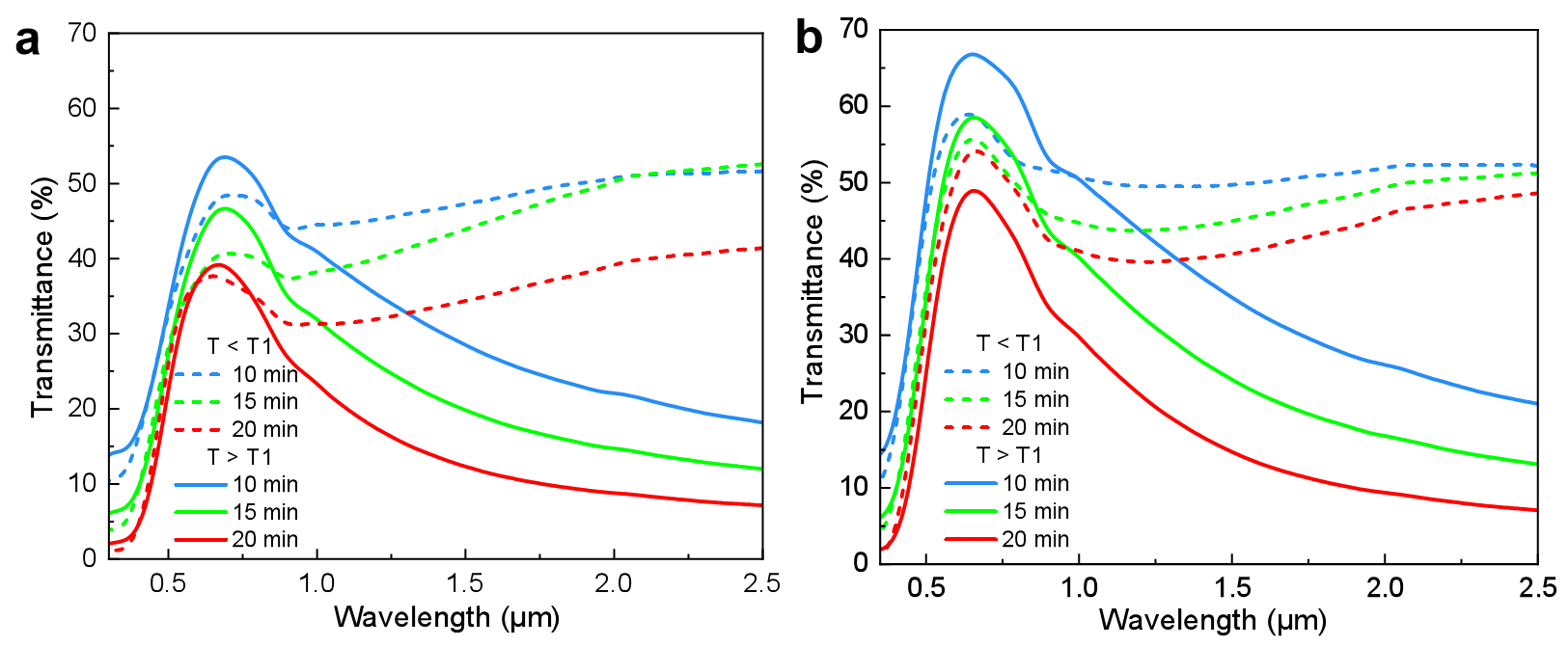


**Figure. S3** UV-Vis-NIR spectra of W-VO_2_ films with different growth times at T < T1 and T > T1.

**
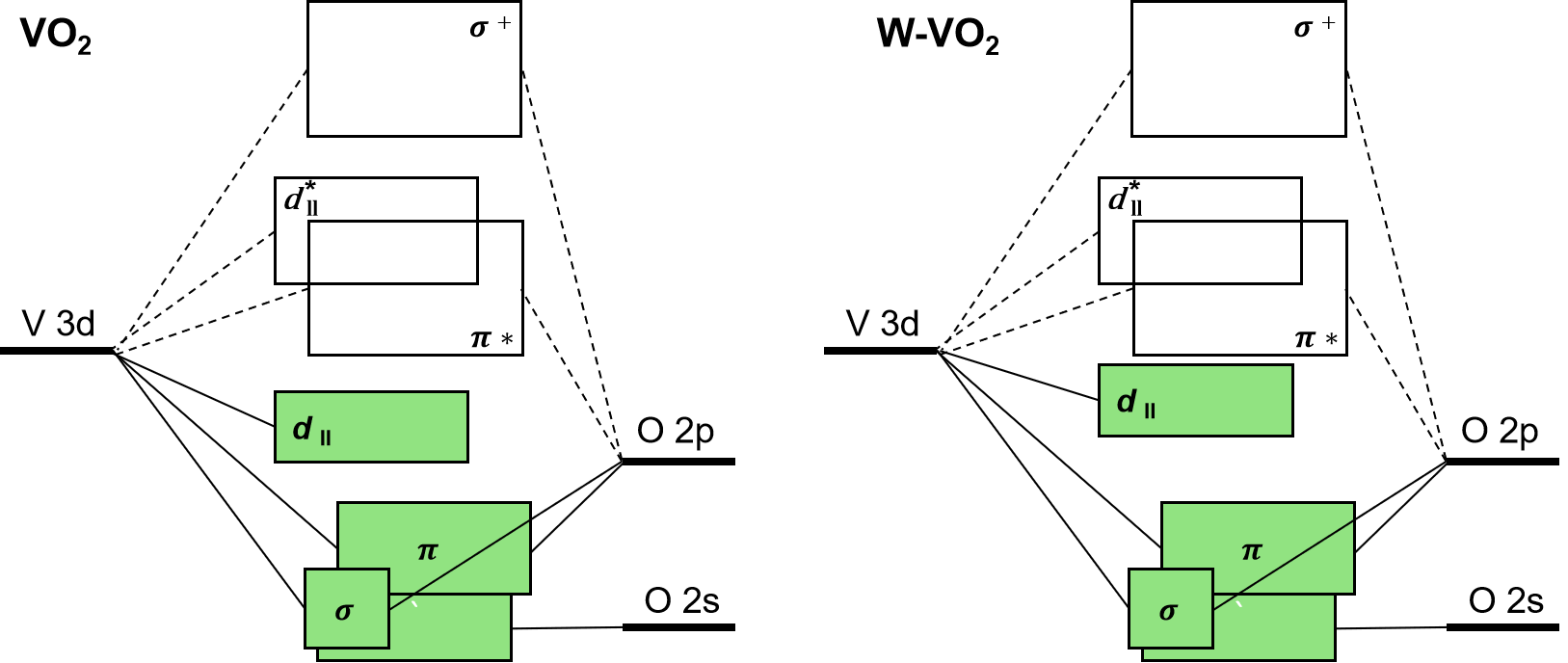
**

**Figure. S4** Monoclinic VO_2_ and W-VO_2_ energy band structure schematic diagrams^[10, 11]^.


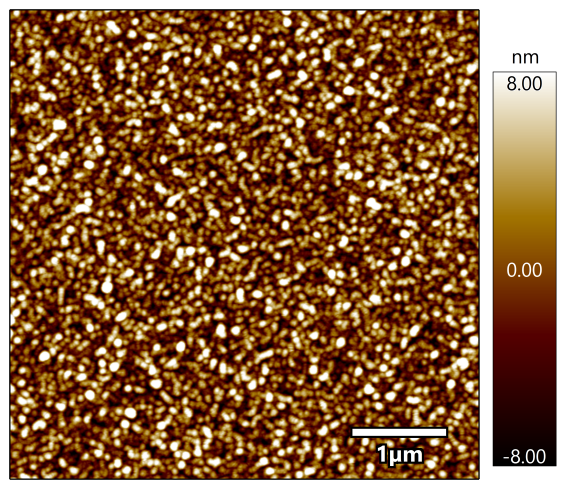


**RMS: 4.097 nm**

**Figure. S5** AFM images VO_2_ thin film, 5 × 5 μm. The dark regions were assumed to represent areas with zero or near zero height values in the positive direction, whereas the bright regions represented higher areas such as the top of bulging grains. The film surface was quite homogeneous and continuous. The RMS surface roughness of the VO_2_ films was detected to be ~ 4.097 nm^[12, 13]^.

**
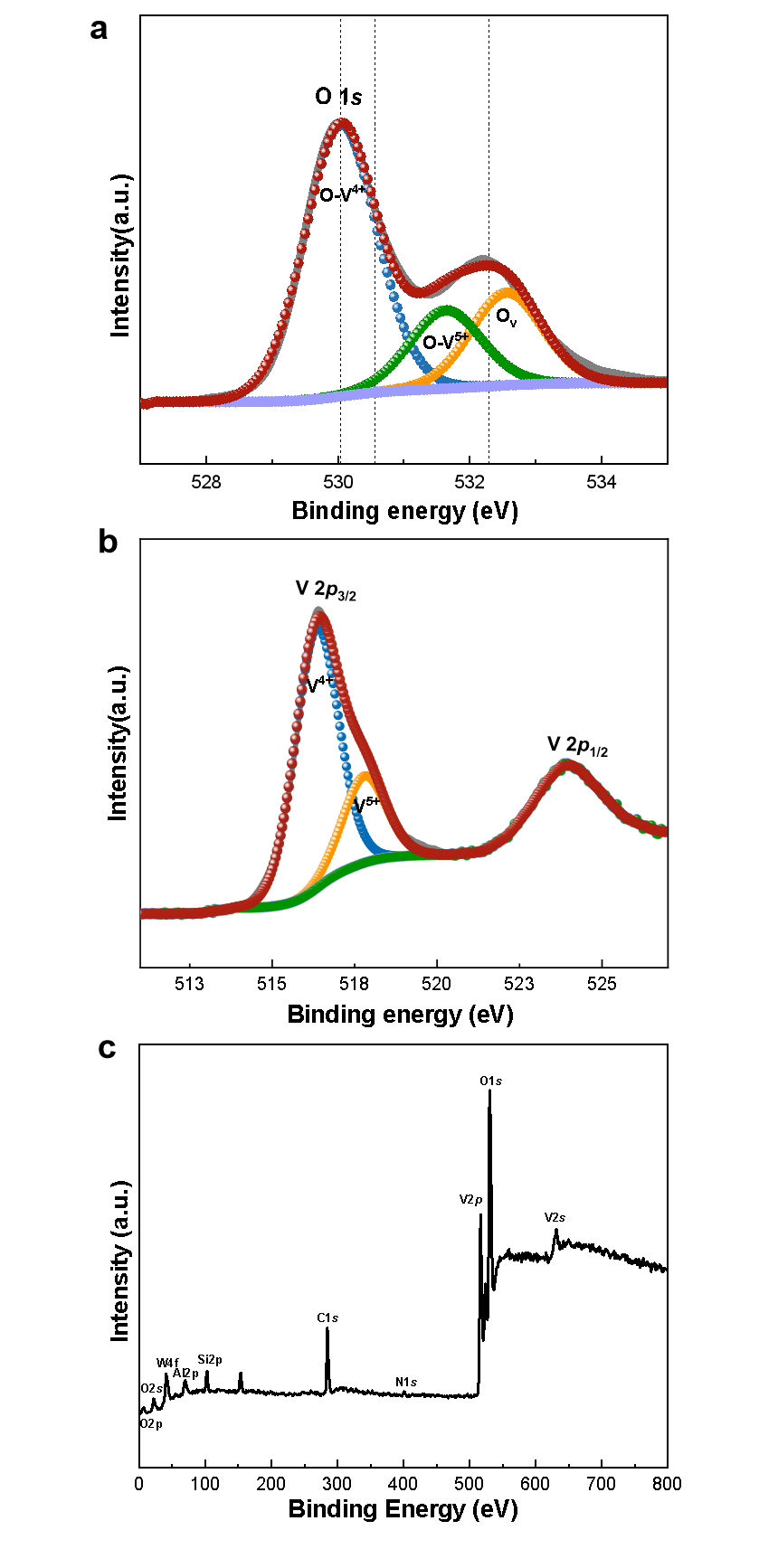
**

**Figure. S6 XPS measurements for the prepared W-VO_2_** **film, the W doping ratio is 1.43%. a)** The curve-fittings of O 1*s* peaks. **b)** The curve-fittings of V 2*p*_3/2_ and V 3*p*_1/2_ peaks. **c)** The full spectrum. The signal was mainly from the V and O, and the C 1s, Si 2*p* and N1*s* peaks may be from surface contaminants or adsorbents.


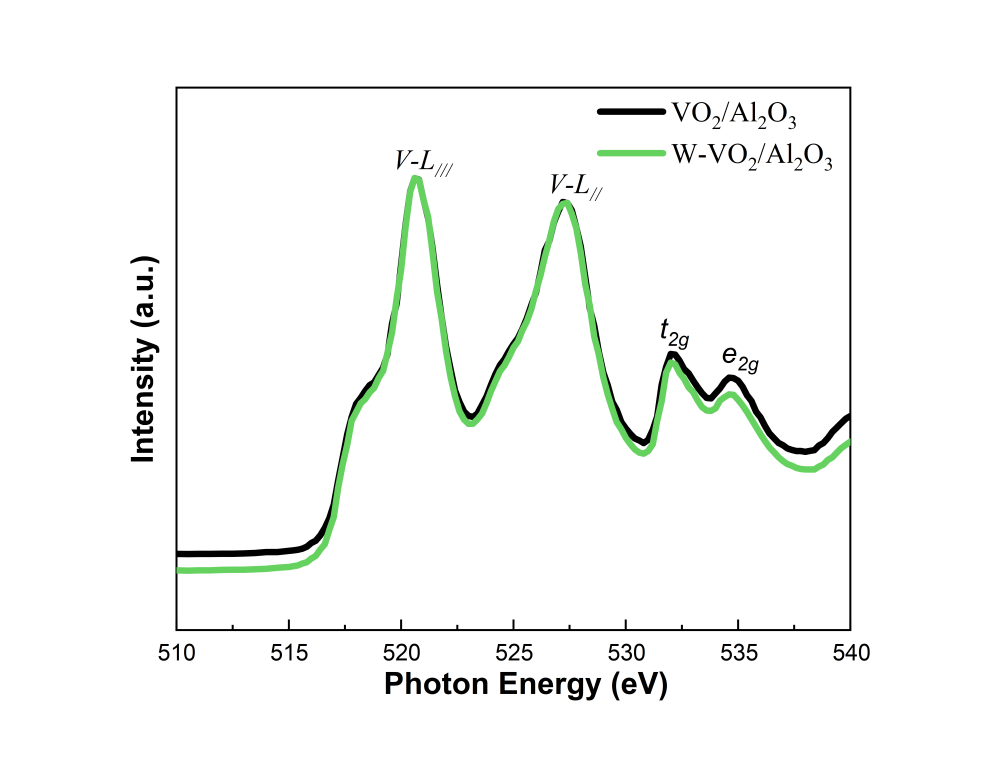


**Figure. S7 The** **XANES curve for VO_2_ and W-VO_2_ thin films. The XANES curves had well-defined V-L fringes at 523.8 eV (2p_1/2_) and 517.6 eV (2p_3/2_), as well as O-K fringes at the e_g_ and t_2g_ peaks. Due to the small proportion of doped W, the peak shift is not significant and only a weak shift to the low-energy region occurs.** The testing was done at Line BL12B station of the National Synchrotron Radiation Laboratory (NSRL), Hefei. The vacuum on the back and bottom of the device was better than 5×10^−8^Pa, the energy resolution was 2000@244 eV (E/ΔE), the light flux was 5×10^9^ phs/s (@244 eV, 300mA), the spot size was 1 mm× 0.5 mm. The leakage current of the sample was collected by Full electron yield mode (TEY), the energy scanning range was 500~536 eV, and the resolution was 0.2 eV.

**
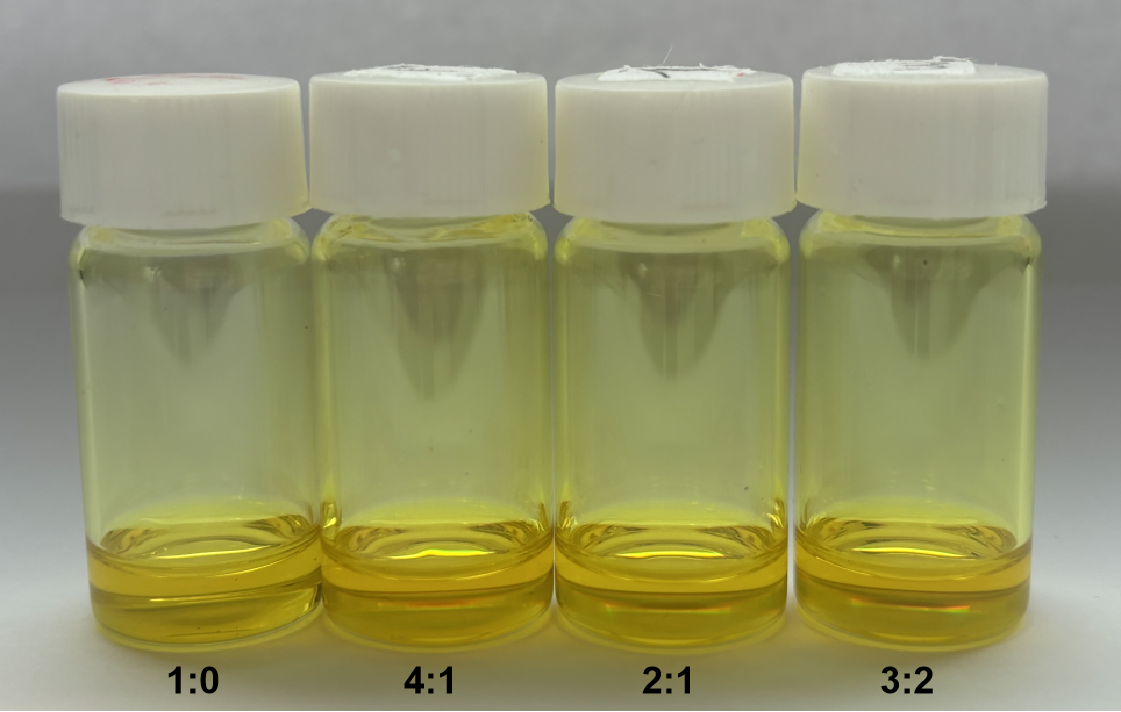
**

**Figure. S8** Photographs of solutions with different mixing ratios (DMF: DMSO).


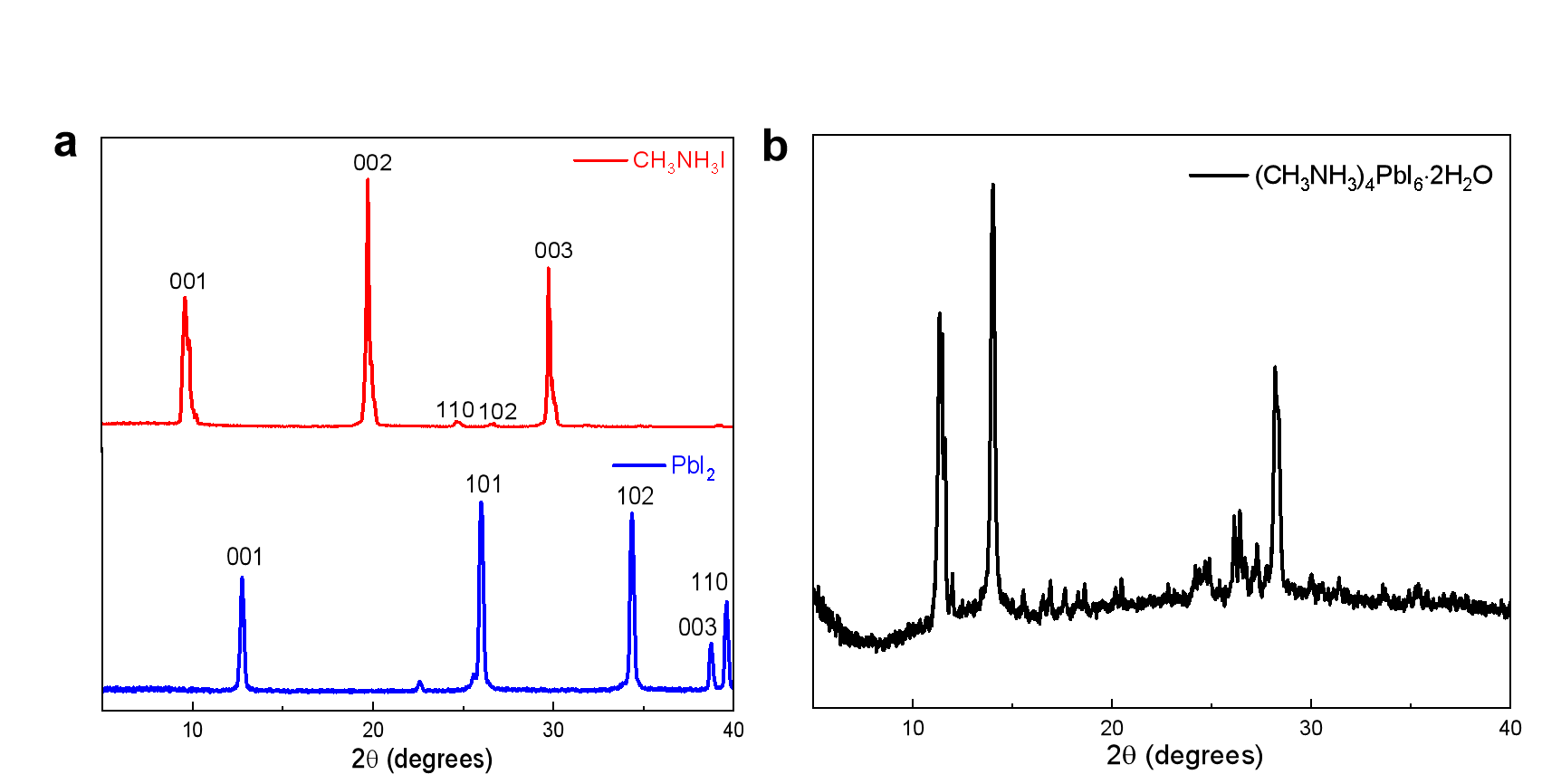


**Figure. S9** XRD patterns of CH_3_NH_3_I, PbI_2,_ and (CH_3_NH_3_)_4_PbI_6_·2H_2_O^[14, 15]^.


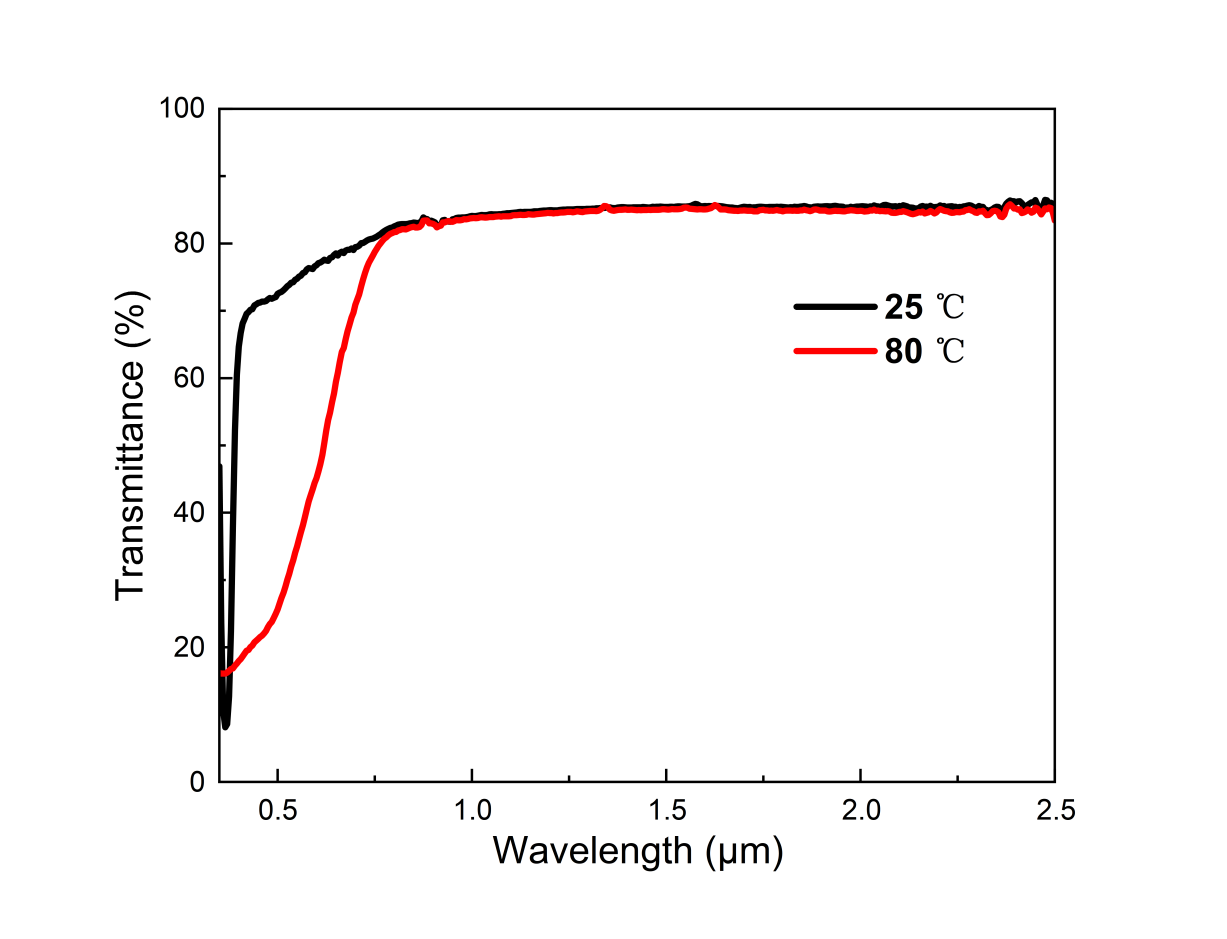


**Figure. S10** Transmittance spectrum of perovskite at the cold and hot states.


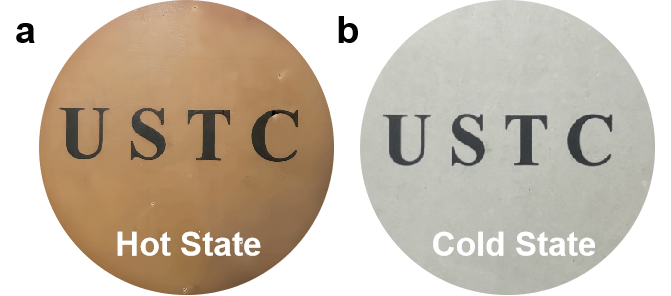


**Figure. S11.** Photograph of **a** hot state (brown colored) and **b** cold state (non-colored) perovskite smart windows. The windows in the hot state are brown and the windows in the cold state are highly transparent.


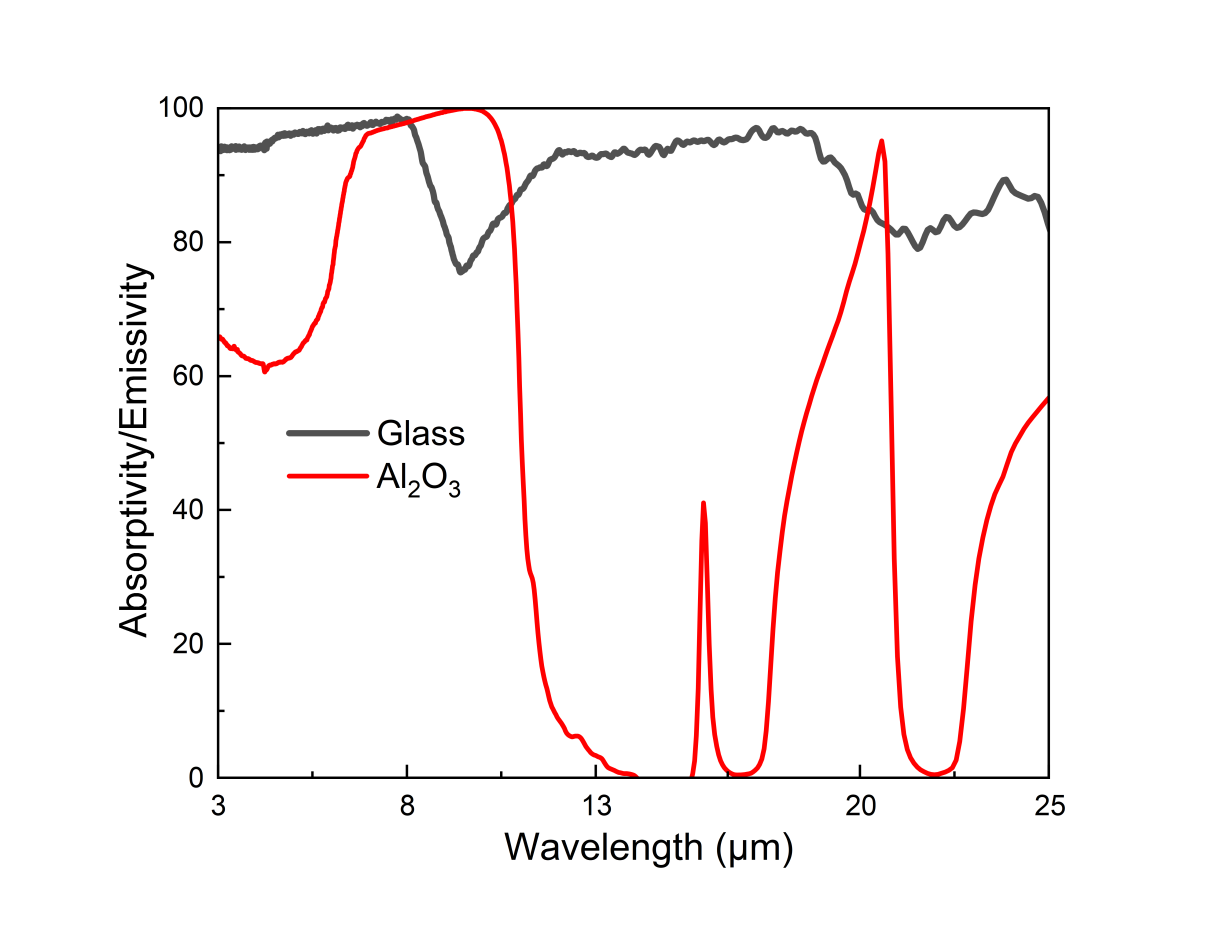


**Figure. S12** MIR emissivity (3-25µm) of glass and Al_2_O_3_ substrate^[16, 17]^.

**
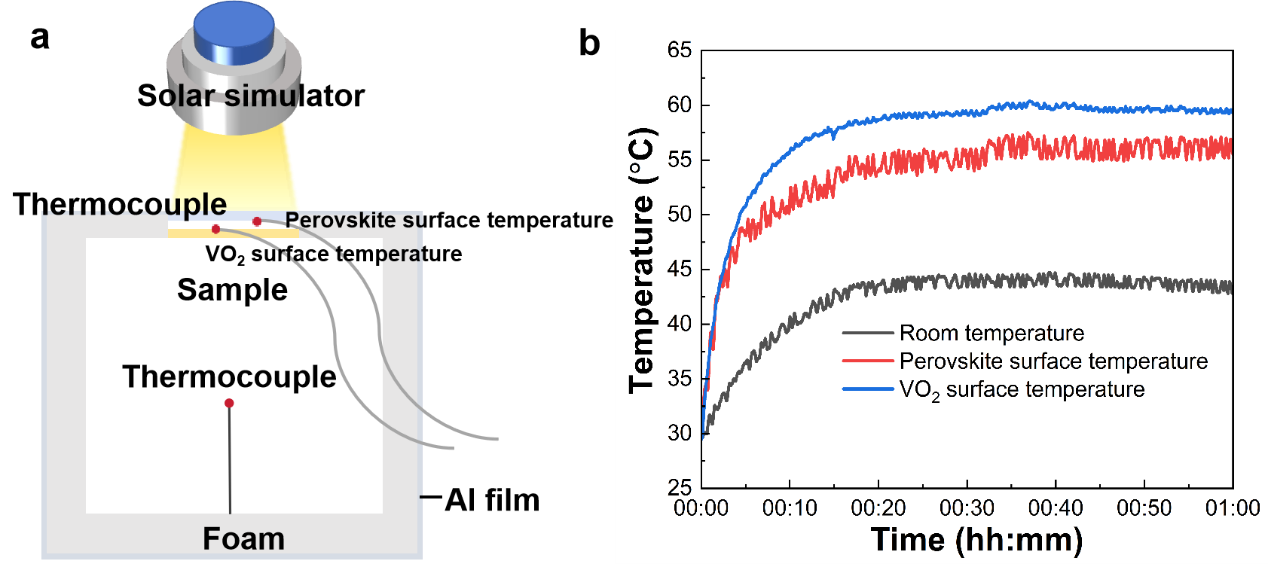
**

**Figure. S13 a)** Experimental setup diagram. **b)** Internal surface temperature profile of Three-State smart window TSSW - To verify whether the three-state is feasible.


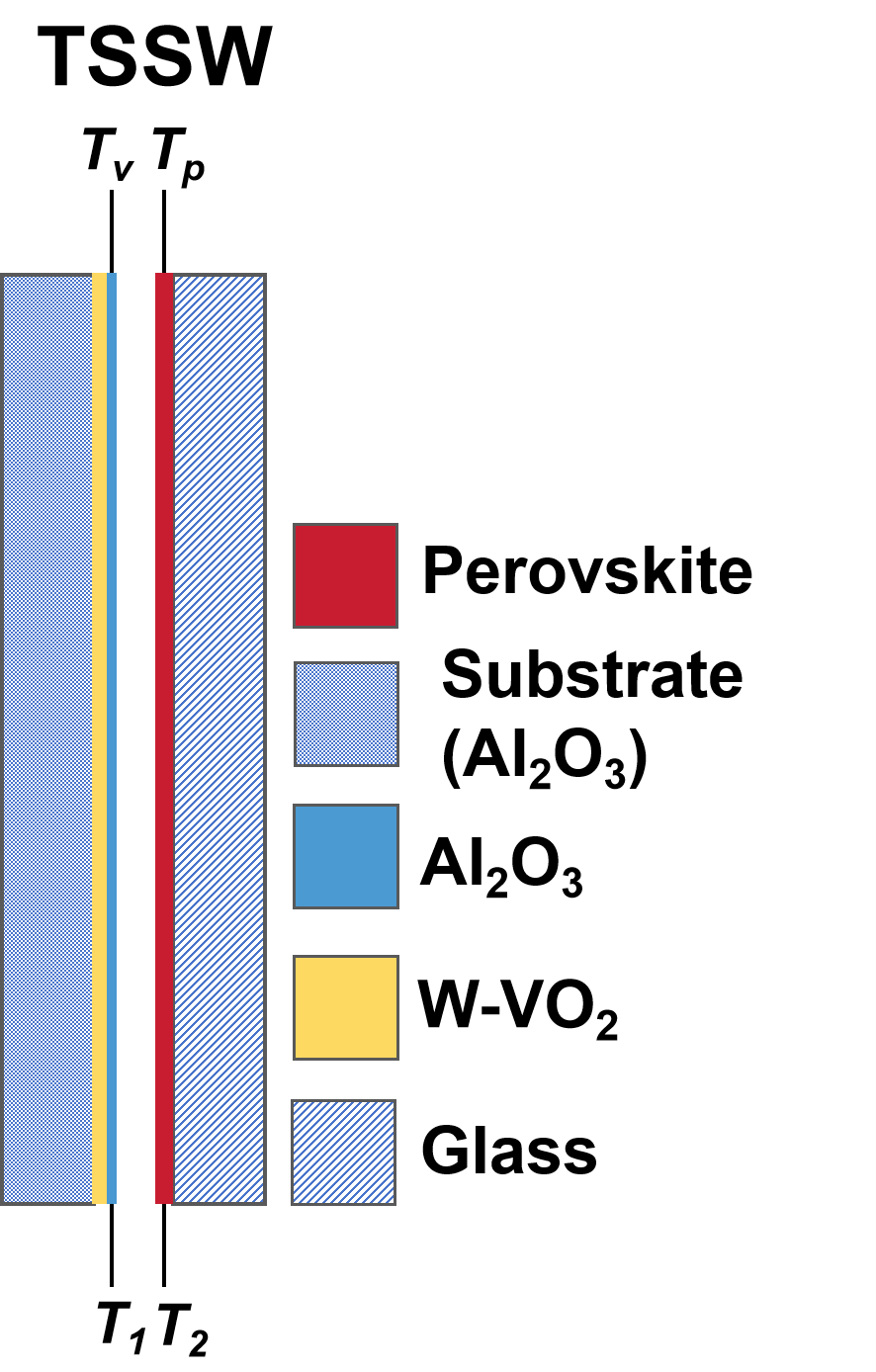


**Figure. S14** Design of relevant temperatures in TSSW.

**
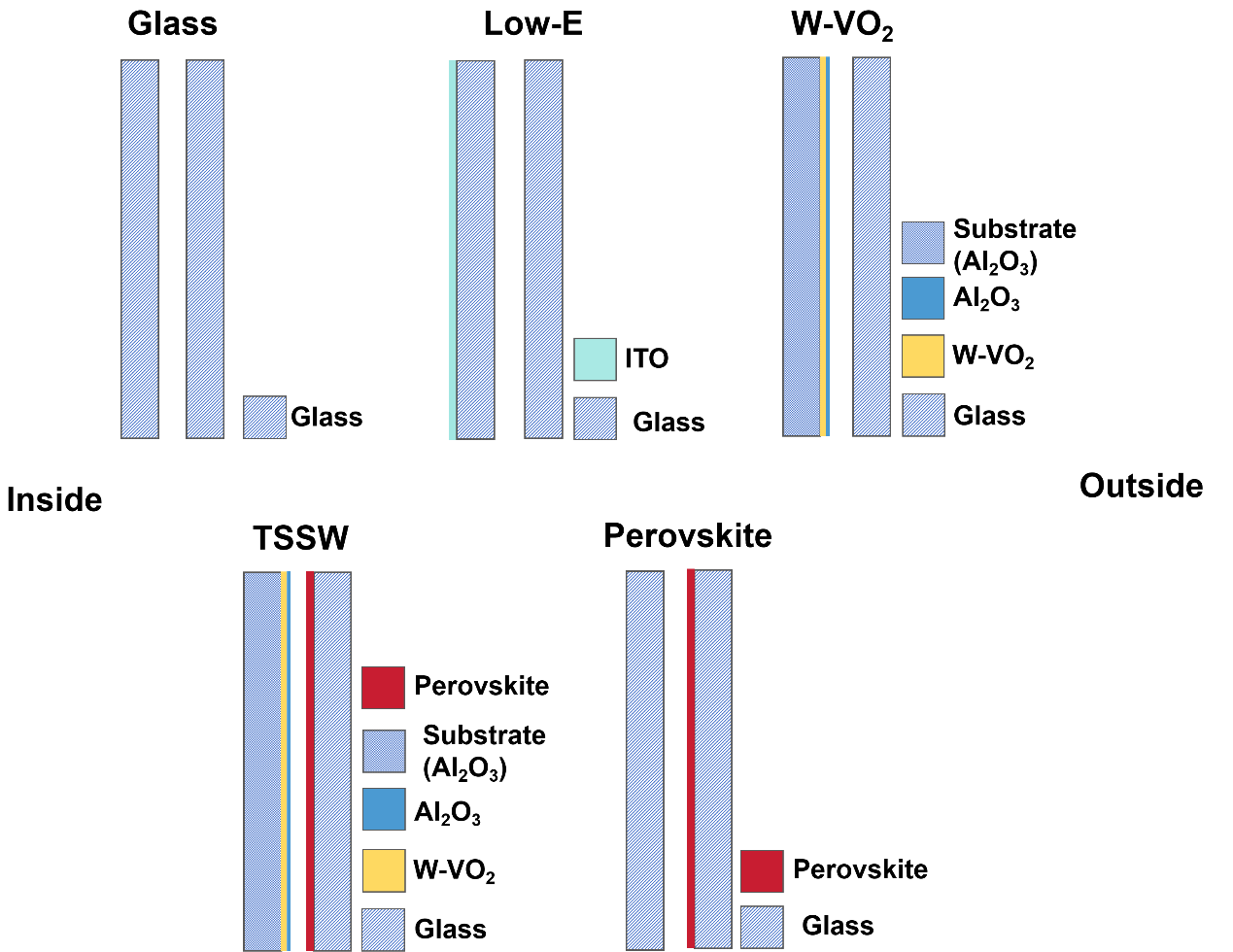
**

**Figure. S15** Design drawings of five window structures used for indoor experiments.

**Figure. S16** 21 h window temperature curve of outside surface for the model house field test on the 24th August 2024 in Hefei.


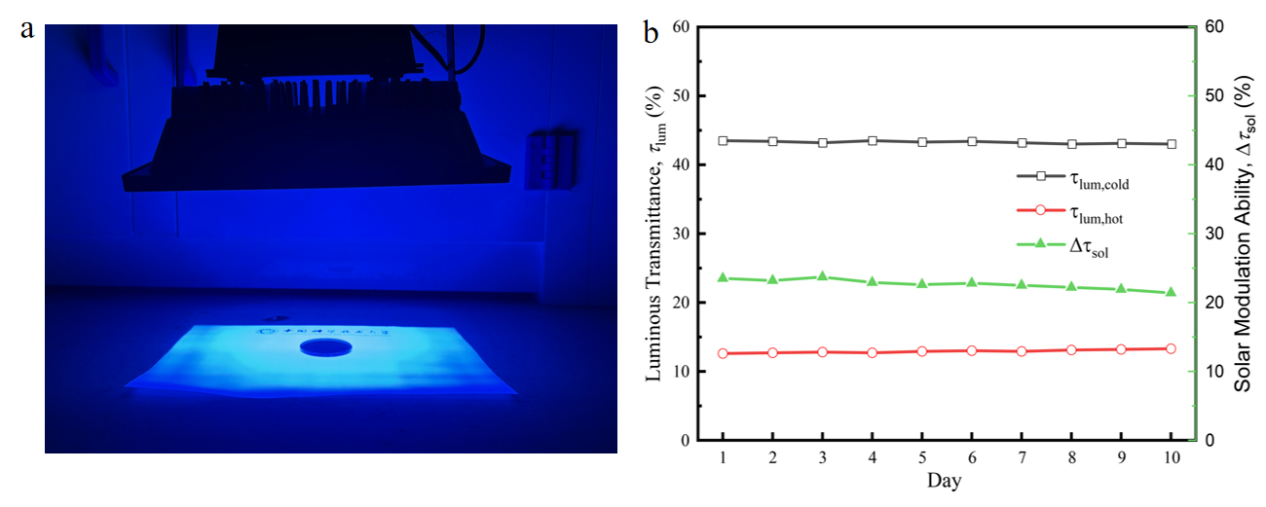
**Figure. S17** Stability testing of the TSSW smart window under UV illumination.


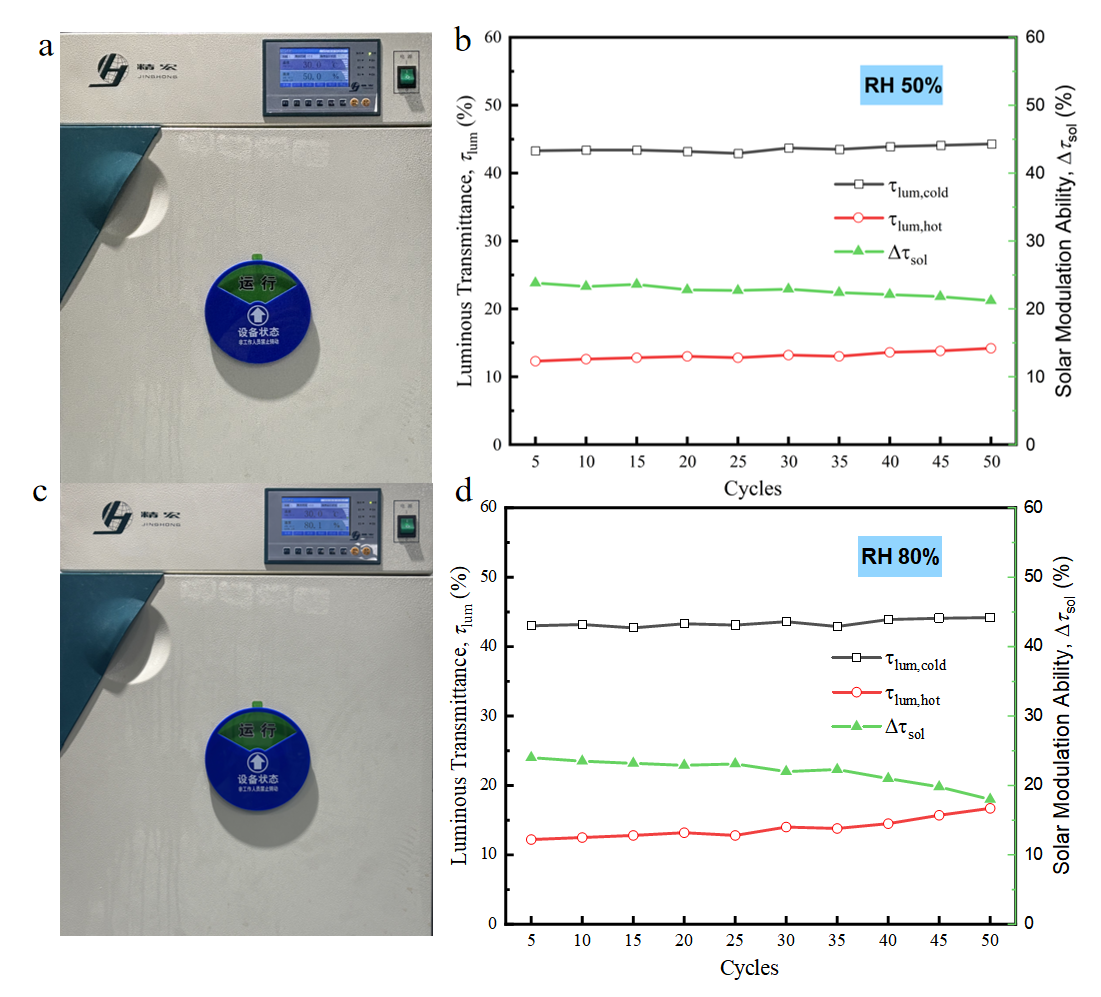


**Figure. S18** Stability test of TSSW smart window at different humidity levels.

**Reference**

[1] Y. Zhou, Y. Cai, X. Hu, Y. Long, *Journal of Materials Chemistry A* **2014**, 2, 13550.

[2] M. Wu, Y. Shi, R. Li, P. Wang, *ACS Appl. Mater. Interfaces* **2018**, 10, 39819.

[3] Y.-S. Yang, Y. Zhou, F. Y. C. Boey, Y. Long, *RSC Adv.* **2016**, 6, 61449.

[4] S. Liu, Y. W. Du, C. Y. Tso, H. H. Lee, R. Cheng, S. P. Feng, K. M. Yu, *Advanced Functional Materials* **2021**, 31, 2010426.

[5] J. Du, Y. Gao, Z. Chen, L. Kang, Z. Zhang, H. Luo, *Sol. Energy Mater. Sol. Cells* **2013**, 110, 1.

[6] X. Qian, N. Wang, Y. Li, J. Zhang, Z. Xu, Y. Long, *Langmuir* **2014**, 30, 10766.

[7] S. Wang, T. Jiang, Y. Meng, R. Yang, G. Tan, Y. Long, *Science* **2021**, 374, 1501.

[8] C. Lin, J. Hur, C. Y. Chao, G. Liu, S. Yao, W. Li, B. Huang, *Science advances* **2022**, 8, eabn7359.

[9] Y. Jiao, Z. Li, C. Li, C. Cao, A. Huang, P. He, X. Cao, *Chem. Eng. J.* **2024**, 497, 154578.

[10] M. Liu, B. Su, Y. Tang, X. Jiang, A. Yu, *Adv. Energy Mater.* **2017**, 7, 1700885.

[11] J. Zhou, M. Xie, A. Cui, B. Zhou, K. Jiang, L. Shang, Z. Hu, J. Chu, *ACS Appl. Mater* **2018**, 10, 30548.

[12] Y. Bleu, F. Bourquard, V. Barnier, A.-S. Loir, F. Garrelie, C. Donnet, *Materials* **2023**, 16, 461.

[13] G. Pan, J. Yin, K. Ji, X. Li, X. Cheng, H. Jin, J. Liu, *Sci. Rep.* **2017**, 7, 6132.

[14] K. Manukyan, A. Yeghishyan, D. Moskovskikh, J. Kapaldo, A. Mintairov, A. Mukasyan, *J. Mater.* **2016**, 51, 9123.

[15] Q. Guo, C. Li, W. Qiao, S. Ma, F. Wang, B. Zhang, L. Hu, S. Dai, Z. a. Tan, *Energy Environ. Sci* **2016**, 9, 1486.

[16] X. Ao, B. Li, B. Zhao, M. Hu, H. Ren, H. Yang, J. Liu, J. Cao, J. Feng, Y. Yang, Z. Qi, L. Li, C. Zou, G. Pei, *Proc. Natl. Acad. Sci. U. S. A.* **2022**, 119, e2120557119.

[17] M. Liu, X. Li, L. Li, L. Li, S. Zhao, K. Lu, K. Chen, J. Zhu, T. Zhou, C. Hu, Z. Lin, C. Xu, B. Zhao, G. Zhang, G. Pei, C. Zou, *Acs Nano* **2023**, 17, 9501.
